# Intestinal epithelial formyl peptide receptor 2 contributes to intestinal epithelium homeostasis and injury repair by regulating intestinal stem cell and transit-amplifying cell proliferation and differentiation

**DOI:** 10.1093/lifemeta/loaf045

**Published:** 2025-12-24

**Authors:** Shuting Yu, Lele Song, Wunier, Shuyu Ouyang, Youpeng Ding, Lixing Zhan, Yi Arial Zeng, Yingying Le

**Affiliations:** Shanghai Institute of Nutrition and Health, University of Chinese Academy of Sciences, Chinese Academy of Sciences, Shanghai 200031, China; Shanghai Institute of Nutrition and Health, University of Chinese Academy of Sciences, Chinese Academy of Sciences, Shanghai 200031, China; Shanghai Institute of Nutrition and Health, University of Chinese Academy of Sciences, Chinese Academy of Sciences, Shanghai 200031, China; Shanghai Institute of Nutrition and Health, University of Chinese Academy of Sciences, Chinese Academy of Sciences, Shanghai 200031, China; Fudan University Shanghai Cancer Center, Fudan University, Shanghai 200032, China; Shanghai Institute of Nutrition and Health, University of Chinese Academy of Sciences, Chinese Academy of Sciences, Shanghai 200031, China; CAS Center for Excellence in Molecular Cell Science, Institute of Biochemistry and Cell Biology, University of Chinese Academy of Sciences, Chinese Academy of Sciences, Shanghai 200031, China; Shanghai Institute of Nutrition and Health, University of Chinese Academy of Sciences, Chinese Academy of Sciences, Shanghai 200031, China

**Keywords:** formyl peptide receptor 2, intestinal epithelium homeostasis, intestinal stem cell, transit-amplifying cell, Wnt pathway, intestinal epithelium injury repair

## Abstract

Intestinal stem cells (ISCs) play critical roles in the self-renewal and regeneration of the intestinal epithelium under physiological conditions and after injury, respectively. However, the underlying mechanisms are not fully understood. In this study, we investigate the role of the G protein-coupled receptor formyl peptide receptor 2 (FPR2) in intestinal epithelium homeostasis and regeneration. In mice, knocking out *Fpr2* in either intestinal epithelial cells (IECs) or ISCs significantly reduces villus height and crypt depth by impairing ISC and transit-amplifying (TA) cell proliferation and differentiation, primarily TA cell differentiation. Mechanistic studies using intestinal organoid culture and bulk and single-cell RNA sequencing revealed that activation of FPR2 promotes proliferation and differentiation of ISCs and TA cells by activating the wingless/integrated (Wnt), Notch, and Hippo signaling pathways via protein kinase C (PKC)−extracellular signal-regulated kinase (ERK). Under physiological conditions, the Wnt and Notch signaling pathways mediate the regulation of ISC proliferation and differentiation by FPR2. *Fpr2* deficiency in mouse IECs exacerbates X-ray- and 5-fluorouracil-induced villus and crypt injury, and delays intestinal epithelium regeneration by reducing ISC and TA cell proliferation. Administering an FPR2 agonist to mice significantly increases survival rates and accelerates intestinal epithelium regeneration after irradiation. Taken together, these results demonstrate that intestinal epithelial FPR2 plays a key role in intestinal epithelium homeostasis and regeneration by promoting ISC and TA cell proliferation and differentiation. FPR2 is a potential therapeutic target against chemotherapy- and radiotherapy-induced intestinal injury.

## Introduction

The intestine is the primary organ responsible for digestion and absorption of nutrients in mammals. The intestinal epithelium renews every 3 to 5 days in most mammals and is responsible for nutrient absorption, energy metabolism, and barrier function [1, 2]. Intestinal stem cells (ISCs), located at the bottom of intestinal crypts, maintain the renewal and function of the intestinal epithelium through self-renewal and differentiation into various intestinal epithelial cell (IEC) types [3, 4]. ISCs are also essential for the regeneration of intestinal epithelium after injury, such as intestinal mucosal injury caused by radiotherapy and chemotherapy for malignant tumors [5, 6]. The fate and function of ISCs under phy­siological conditions and after intestinal mucosal injury are regulated by a variety of microenvironmental factors, such as wingless/integrated (Wnt), Notch ligands, epidermal growth factor, bone morphogenetic protein, and R-spondins, produced by Paneth cells and mesenchymal cells [3, 7, 8]. The gut microbiota and its products are also involved in the regulation of ISC proliferation and differentiation [9, 10]. ISCs express the pattern recognition receptors (PRRs) including Toll-like receptor 4 (TLR4) and nucleotide-binding oligomerization domain-containing protein 2 (NOD2) [11, 12]. The TLR4 agonist lipopolysaccharide from crypt-specific core microbiota inhibits ISC proliferation, increases ISC apoptosis, and enhances goblet cell lineage differentiation [11, 13]. TLR4 regulation of ISCs contributes to the pathogenesis of necrotizing enterocolitis [11]. Gut microbiota-derived NOD2 agonist protects ISCs from oxidative stress-mediated cell death and promotes intestinal epithelial regeneration [12, 14]. NOD2 supports intestinal epithelial regene­ration after radiation-induced intestinal epithelium injury [15]. These results suggest that activation of PRRs by bacterial products leads to distinct fates of ISCs. Investigating whether other PRRs contribute to the regulation of ISC fate under physiological conditions and after intestinal epithelium injury will provide new insights into the mechanisms involved in intestinal epithelium homeostasis and identify potential targets for the treatment of intestinal epithelium injury.

Formyl peptide receptor 2 (FPR2) is a G protein-coupled receptor that belongs to the FPR family [16, 17]. Mouse FPR2 is a homolog of human FPR2 [18]. FPR2 is a PPR that recognizes a variety of ligands, including bacterial and viral peptides, host-produced peptides and lipids, synthetic peptides, and small molecules [16, 17, 19]. It is expressed in phagocytic leukocytes, epithelial cells, endothelial cells, hepatocytes, and glial cells, etc. [16, 20, 21]. FPR2 has been reported to be involved in host defense and wound healing, as well as in pathological conditions such as inflammation-related diseases and tumor growth [16, 17, 20, 22–25], with most studies focusing on leukocyte-expressed FPR2. Our previous collaborative studies showed that systemic *Fpr2* knockout in mice results in reduced colonic crypt depth and acute inflammatory responses, and delayes mucosal recovery from dextran sulfate sodium (DSS)-induced injury [20]. These results indicate that FPR2 may contribute to colonic epithelial homeostasis, inflammation, and colonic epithelium regeneration after injury. However, whether FPR2 expressed by IECs plays a critical role in intestinal epithelium homeostasis under physiological conditions and regeneration after injury is unknown.

In this study, we investigated the contribution of intestinal epithelial FPR2 to intestinal epithelium homeostasis under physiological conditions and regeneration after X-ray irradiation (IR) and 5-FU-induced intestinal epithelium injury using IEC-specific and ISC-specific *Fpr2* knockout mice and explored the underlying mechanisms. We report that intestinal epithelial FPR2 is required for intestinal epithelium homeostasis and regeneration by regulating ISC and transit-amplifying (TA) cell proliferation and differentiation.

## Results

### Intestinal epithelial *Fpr2* deficiency impairs intestinal epithelium homeostasis in mice

**Figure 1:**
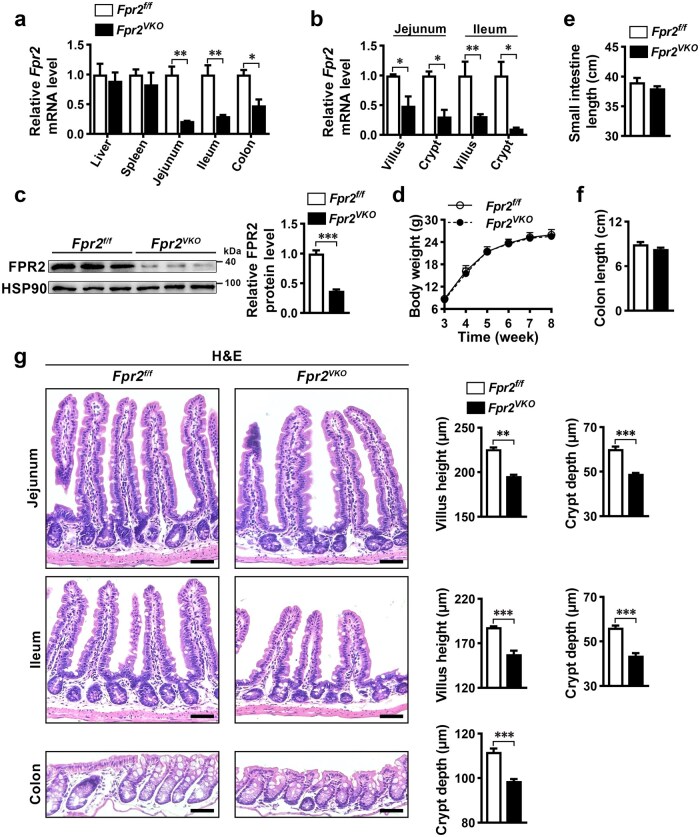
Intestinal epithelial *Fpr2* deficiency in mice impairs intestinal epithelium homeostasis. (a and b) Expression of *Fpr2* mRNA in tissues and organs of IEC-specific *Fpr2* knockout mice (*Fpr2^VKO^*) and control mice *(Fpr2^f/f^*) determined by RT-qPCR. *n *= 3 mice/group. (c) Expression of FPR2 protein in ileal organoids derived from *Fpr2^f/f^* and *Fpr2^VKO^* mice determined by western blot analysis. *n *= 3 mice/group. (d) Body weight of *Fpr2^f/f^* and *Fpr2^VKO^* mice after birth. *n *= 10 mice/group. (e and f) Length of the small intestine (e) and colon (f) in *Fpr2^f/f^* and *Fpr2^VKO^* mice. *n *= 8 mice/group. (g) Representative H&E staining images (left) of jejunal, ileal, and colonic sections from *Fpr2^f/f^* and *Fpr2^VKO^* mice, with quantification of villus height and crypt depth (right). *n *= 6 mice/group. Scale bar, 50 μm. Data are presented as mean ± SEM. ^*^P < 0.05; ^**^P < 0.01; ^***^P < 0.001, by unpaired two-tailed Student’s *t* test.

Intestinal epithelium-specific *Fpr2* knockout (*Fpr2^VKO^*) mice were generated to investigate the involvement of FPR2 in intestinal epithelium homeostasis. Reverse transcription-quantitative polymerase chain reaction (RT-qPCR) analysis revealed that *Fpr2* mRNA levels were significantly reduced in the jejunum, ileum, and colon, as well as in the villi and crypts isolated from the jejunum and ileum, but did not alter in the liver and spleen in *Fpr2^VKO^* mice compared to control *Fpr2^f/f^* mice (Fig. 1a and b). Western blot ana­lysis revealed that FPR2 protein levels were significantly lower in ileal organoids derived from *Fpr2^VKO^* mice than in organoids derived from *Fpr2^f/f^* mice (Fig. 1c). These results indicate that *Fpr2* is speci­fically deleted in the intestinal epithelium. Body weight and length of small intestine and colon were not different between *Fpr2^VKO^* and *Fpr2^f/f^* mice (Fig. 1d–f). Hematoxylin and eosin (H&E) staining showed that villus height and crypt depth in the small intestine and crypt length in the colon were significantly reduced in *Fpr2^VKO^* mice (Fig. 1g). These results indicate that intestinal epithelial FPR2 plays an important role in maintaining intestinal epithelium homeostasis.

### *Fpr2* deficiency in mouse intestinal epithelium reduces several epithelial cell types and crypt cell proliferation

**Figure 2:**
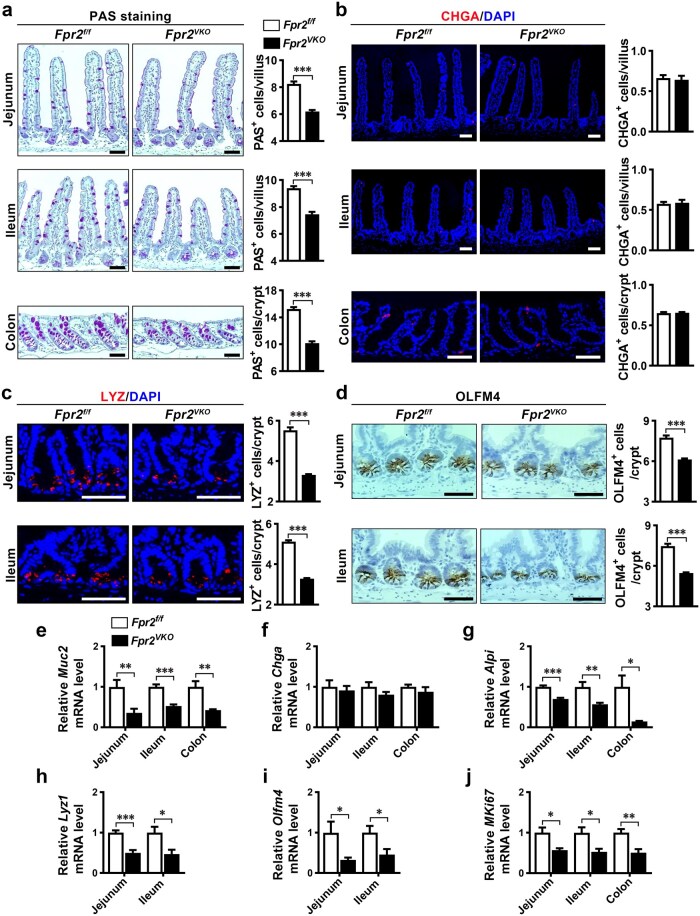
Alteration of IECs in intestinal epithelial *Fpr2*-deficient mice. (a) Representative PAS staining images of jejunal, ileal, and colonic sections from *Fpr2^f/f^* and *Fpr2^VKO^* mice, with quantification of positive cells. (b–d) Representative images of immunofluorescence staining for CHGA (b) and LYZ (c), and immunohistochemical staining for OLFM4 (d) in sections of jejunum, ileum, and colon from *Fpr2^f/f^* and *Fpr2^VKO^* mice, with quantification of positive cells. (e–j) The expression of various IEC type marker genes and the cell proliferation marker gene *Mki67* determined by RT-qPCR in small intestinal tissues of *Fpr2^f/f^* and *Fpr2^VKO^* mice. *n *= 6 mice/group. Scale bar, 50 μm. Data are presented as mean ± SEM. ^*^P < 0.05; ^**^P < 0.01; ^***^P < 0.001, by unpaired two-tailed ­Student’s *t* test.

We investigated the effect of intestinal epithelial *Fpr2* deficiency on IECs in mice. Periodic acid-Schiff (PAS) staining revealed a significant decrease in PAS^+^ goblet cells in the small intestinal villi and colonic crypts of *Fpr2^VKO^* mice compared to *Fpr2^f/f^* mice (Fig. 2a). Immunofluo­rescence staining for chromogranin A (CHGA) revealed that CHGA^+^ enteroendocrine (EE) cells in the small intestinal villi and colonic crypts were not different between *Fpr2^VKO^* and *Fpr2^f/f^* mice (Fig. 2b). Immunofluorescence staining for lysozyme (LYZ) and immunohistochemical staining for olfactomedin 4 (OLFM4) revealed a significant decrease in LYZ^+^ Paneth cells (Fig. 2c) and OLFM4^+^ ISCs (Fig. 2d) in the crypts of the jejunum and ileum in *Fpr2^VKO^* mice. However, the distribution of different IEC types in the intestinal villi and crypts was not different between *Fpr2^VKO^* and *Fpr2^f/f^* mice (Fig. 2a–d). RT-qPCR analysis for the expression of marker genes for goblet cells (*Muc2*), EE cells (*Chga*), enterocytes (*Alpi*), Paneth cells (*Lyz1*), ISCs (*Olfm4*), and a cell proliferation marker (*Mki67*) in the small intestine demonstrated a significant reduction in the mRNA levels of these genes, with the exception of *Chga*, in *Fpr2^VKO^* mice compared with *Fpr2^f/f^* mice (Fig. 2e−j). Collectively, these results demonstrate that *Fpr2* deficiency decreases the number of goblet cells, Paneth cells, enterocytes, and ISCs, and inhibits cell proliferation, which leads to a decrease in villus height and crypt depth in the small intestine and colon of *Fpr2^VKO^* mice.

**Figure 3:**
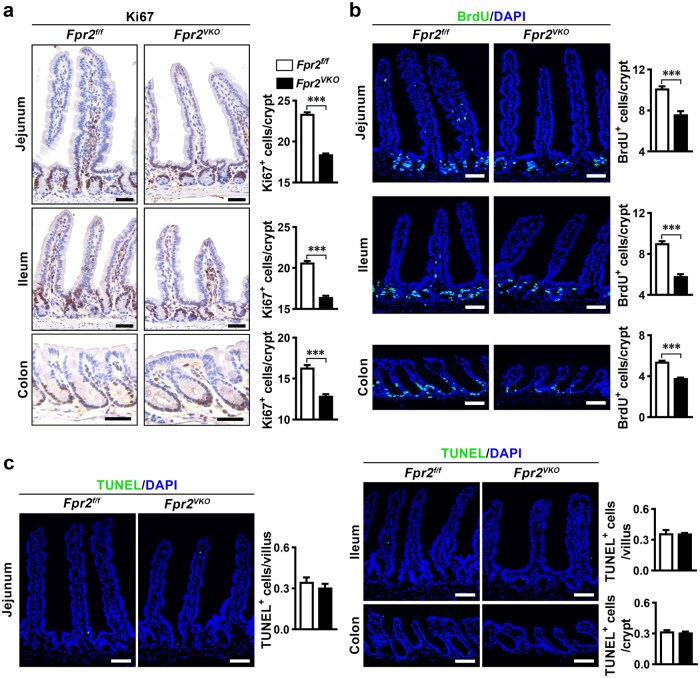
Fpr2 deficiency in mouse intestinal epithelium impairs cell proliferation in intestinal crypts. (a) Representative images of immunohistochemical staining for Ki67 in sections of jejunum, ileum, and colon from *Fpr2^f/f^* and *Fpr2^VKO^* mice, with quantification of positive cells. *n *= 6 mice/group. (b) Representative images of immunofluorescence staining for BrdU in sections of jejunum, ileum, and colon after intraperitoneal injection of BrdU for 2 h in *Fpr2^f/f^* and *Fpr2^VKO^* mice, with quantification of positive cells. *n *= 6 mice/group. (c) Representative images of TUNEL assays of jejunal, ileal, and colonic sections from *Fpr2^f/f^* and *Fpr2^VKO^* mice, with quantification of TUNEL^+^ apoptotic cells (green). *n *= 4 mice/group. Scale bar, 50 μm. Data are presented as mean ± SEM. ^***^P < 0.001, by unpaired two-tailed Student’s *t* test.

We then examined Ki67 expression, 5′-bromo-2′-deoxyuridine (BrdU) incorporation, and apoptotic cells in sections of the intestine via immunostaining and the terminal deoxynucleotidyl transferase (TdT)-mediated dUTP nick-end labelling (TUNEL) assay. The results showed that Ki67^+^ cells were mainly located in the crypts of the small intestine and colon, and were significantly reduced in *Fpr2^VKO^* mice compared to *Fpr2^f/f^* mice (Fig. 3a). The change in BrdU^+^ cells in *Fpr2^VKO^* mice was similar to that of Ki67^+^ cells (Fig. 3b). These results indicate that intestinal epithelial *Fpr2* deficiency inhibits cell proliferation in the intestinal crypts. Since ISCs and TA cells are the only proliferative cells in the crypt, the above results suggest that *Fpr2* deficiency in these cells impairs their proliferation. TUNEL assay showed that there were very few apoptotic cells in the intestine of *Fpr2^f/f^* mice, and *Fpr2* deficiency in the intestinal epithelium had no significant effect on the apoptosis of IECs (Fig. 3c).

### Mice with *Fpr2* deficiency in ISCs exhibit intestinal epithelial phenotypes similar to *Fpr2^VKO^* mice

Since all types of IECs are derived from the differentiation of ISCs, we investigated whether FPR2 in ISCs contributes to intestinal epithelium homeostasis by tamoxifen-induced deletion of *Fpr2* in ISCs in mice (*Lgr5-Fpr2^KO^*). Compared to control *Lgr5-Fpr2^f/f^* mice, *Fpr2* mRNA levels were significantly lower in the jejunum, ileum, and colon, as well as in ISCs isolated from the small intestine in *Lgr5-Fpr2^KO^* mice (Supplementary Fig. S1a and b). However, body weight and the length of the small intestine and colon were not altered in *Lgr5-Fpr2^KO^* mice (Supplementary Fig. S1c–e). H&E staining, PAS staining, TUNEL assays, and immunostaining for Ki67, BrdU, and IEC biomarkers demonstrated that intestinal villus height, crypt depth (Supplementary Fig. S1f), numbers of goblet cells, Paneth cells, and ISCs in the intestine (Supplementary Fig. S2a, c, and d), and numbers of Ki67^+^ and BrdU^+^ cells in the crypts (Supplementary Fig. S3a and b) were all significantly reduced in *Lgr5-Fpr2^KO^* mice. However, EE cells and apoptotic cells in the intestine (Supplementary Figs. S2b and S3c) were not altered in *Lgr5-Fpr2^KO^* mice compared with *Lgr5-Fpr2^f/f^* mice. Taken together, the intestinal phenotypes of *Lgr5-Fpr2^KO^* mice were similar to those of *Fpr2^VKO^* mice, suggesting that *Fpr2* deficiency in ISCs impairs intestinal epithelium homeostasis by reducing ISC and TA cell proliferation.

### FPR2 promotes ISC proliferation and TA cell differentiation

**Figure 4:**
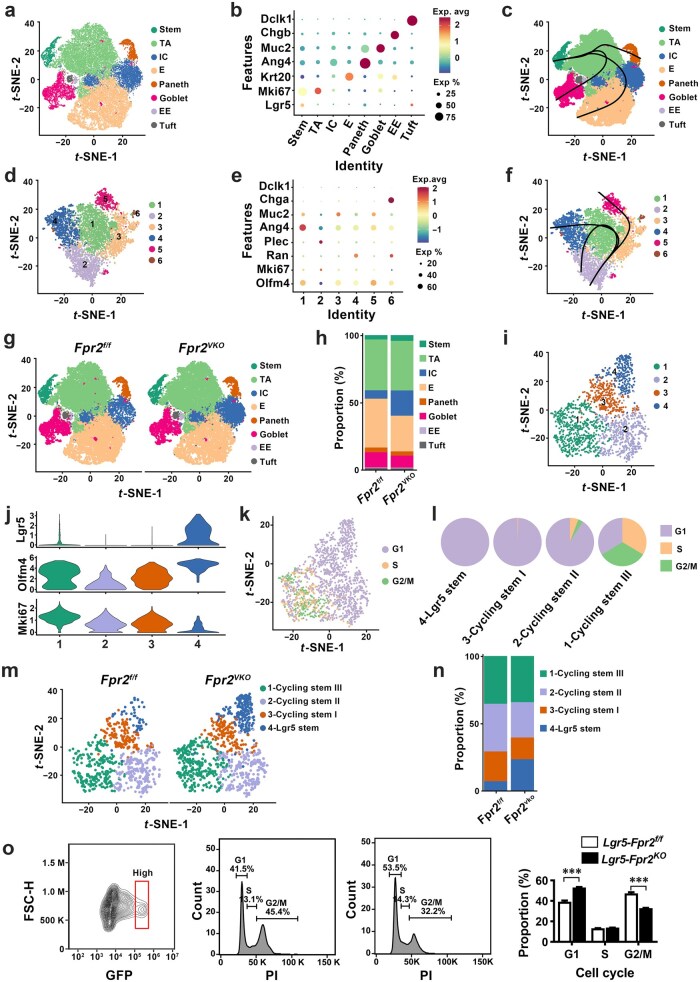
scRNA-seq of IECs from small intestinal crypts reveals aberrant ISC proliferation and differentiation in intestinal epithelial *Fpr2*-deficient mice. (a and b) *t*-SNE visualization of clustering of single epithelial cells isolated from small intestinal crypts (23,078 cells from *Fpr2^f/f^* mice, *n *= 3; 23,444 cells from *Fpr2^VKO^* mice, *n *= 3) (a), and expression of IEC marker genes in each cluster (b). (c) Pseudotime trajectory analysis of all IECs. (d and e) *t*-SNE visua­lization of IC subclusters in intestinal crypts from *Fpr2^f/f^* and *Fpr2^VKO^* mice (d), and expression of IEC marker genes in each subcluster (e). (f) Pseudotime trajectory analysis of subclusters from (d). (g and h) *t*-SNE visualization of clustering of IECs in *Fpr2^f/f^* and *Fpr2^VKO^* mice (g) and percentage of each cluster (h). (i and j) *t*-SNE visualization of stem cell subclusters in *Fpr2^f/f^* and *Fpr2^VKO^* mice (i) and expression of stem cell marker genes and cell proliferation marker *Mki67* in each subcluster (j). (k and l) *t*-SNE visualization of cell cycle phase distribution in the stem cell cluster from *Fpr2^f/f^* and *Fpr2^VKO^* mice (k) and percentage of cells in G1, G2/M, and S phases in each stem cell subcluster (l). (m and n) *t*-SNE visualization of stem cell subclusters in *Fpr2^f/f^* and *Fpr2^VKO^* mice (m) and percentage of each subcluster (n). (o) Analysis of cell cycles and quantification for cell cycle distribution in small intestinal ISCs from *Lgr5-Fpr2^f/f^* and *Lgr5*-*Fpr2^KO^* mice. IECs were isolated from the small intestinal crypts of *Lgr5-Fpr2^f/f^* and *Lgr5*-*Fpr2^KO^* mice. GFP^high^ ISCs were gated to detect DNA content via flow cytometry and quantified for cell cycle distribution. Stem, intestinal stem cells; TA, transit-amplifying cells; IC, immature cells; E, enterocyte cells; EE, enteroendocrine cells; Tuft, tuft cells.

To elucidate the mechanisms of intestinal epithelial FPR2-mediated ISC proliferation regulation, single-cell RNA sequencing (scRNA-seq) was performed on IECs isolated from small intestinal crypts of *Fpr2^f/f^* and *Fpr2^VKO^* mice. Eight distinct cell clusters were identified according to known marker genes for IECs (Fig. 4a and b), including stem cells, TA cells, immature cells (ICs), enterocytes, Paneth cells, goblet cells, EE cells, and tuft cells. Pseudotime analysis of all IECs showed the differentiation trajectories of ISCs−TA cells−mature IECs (Fig. 4c), indicating the accuracy of the cell annotation. With the exception of the IC cluster, which has not been reported previously, the transcriptional profiles of the other cell clusters were similar to published datasets [26]. Further clustering analysis of the ICs identified six subclusters (Fig. 4d) expressing the stem cell marker *Olfm4* and the TA cell markers *Mki67* and *Ran*, as well as low levels of marker genes of various IEC types (Fig. 4e). Annotation using IEC marker genes could not classify any of them into a specific epithelial type. Pseudotime analysis of ICs revealed three differentiation trajectories that were similar to the pathways by which TA cells differentiated into mature IECs [27] (Fig. 4f). These results suggest that the IC cluster comprised heterogeneous subpopulations of immature IECs at various stages of differentiation from TA cells to IECs. Analysis of the scRNA-seq data revealed that several genes involved in cell proliferation (*Peak1*, *Il31ra*, *Ccnb1ip1*, and *Cd44*) [28–31] and differentiation (*Hes1*, *Cdk8*, and *Lars2*) [32–34] were among the top 20 downregulated genes in ICs from *Fpr2^VKO^* mice. The expression of ribosomal protein genes involved in protein synthesis (*Rpl10*, *Rpl11*, *Rpl15*, *Rpl35*, *Rpl36*, *Rps6*, and *Rps11*) was also reduced in ICs from *Fpr2^VKO^* mice (Supplementary Fig. S4a). These results suggest that *Fpr2* deficiency inhibits IC proliferation and differentiation by downregulating genes associated with protein synthesis, and cell proliferation and differentiation. Additionally, *Olfm4*, an ISC marker gene, was expressed at higher levels in ICs from *Fpr2^VKO^* mice than in ICs from *Fpr2^f/f^* mice (Supplementary Fig. S4b), demonstrating that *Fpr2* deficiency inhibits IC differentiation.

Among the eight clusters of IECs, *Fpr2* deficiency reduced enterocytes, TA cells, Paneth cells, goblet cells, and tuft cells, and significantly increased ICs (Fig. 4g and h). These results indicate that *Fpr2* deficiency primarily impairs the terminal differentiation of TA cells, leading to an increase in ICs and a decrease in mature IECs. Since the stem cell cluster was slightly increased in *Fpr2^VKO^* mice compared to *Fpr2^f/f^* mice (Fig. 4h), which seemed to contradict the results of immunohistochemical staining of OLFM4 stem cells (Fig. 2d), the stem cell cluster was further subclustered into four unique clusters based on transcriptional profiles (Fig. 4i). Subcluster 4 cells exhibited high expression of ISC markers (*Lgr5* and *Olfm4*) and concomitantly low levels of the cell proliferation marker *Mki67*, whereas cells in the other subclusters expressed varying levels of *Olfm4* and *Mki67*, but very low levels of *Lgr5* (Fig. 4j), indicating that the stem cell cluster is composed of cells with different proliferative capacities. Cell cycle analysis of the stem cell cluster revealed that most of the cells were in G1 phase and fewer cells were in S and G2/M phases (Fig. 4k). All cells in subcluster 4 were in G1 phase, and the percentage of cells in S and G2/M phases was progressively increased in subclusters 3, 2, and 1 (Fig. 4l). Therefore, we designated subclusters 4, 3, 2, and 1 as Lgr5 stem cells and cycling stem cells I−III, respectively. There was an increase in Lgr5 stem cells and a decrease in cycling stem cells I, II, and III in *Fpr2^VKO^* mice (Fig. 4m and n). These results indicate that deletion of intestinal epithelial *Fpr2* inhibits stem cell proliferation by blocking cell cycle progression from G1 to S and G2/M phases. We further analyzed the cell cycles of small intestinal ISCs from *Lgr5-Fpr2^f/f^* and *Lgr5*-*Fpr2^KO^* mice via flow cytometry. The results revealed that *Fpr2* deficiency in ISCs significantly increased the percentage of cells in the G1 phase and decreased the percentage of cells in the G2/M phase (Fig. 4o). These results confirm that *Fpr2* deficiency inhibits ISC proliferation by blocking progression of cell cycle from the G1 to S and G2/M phases.

We further analyzed the transcriptional profiles of the stem cell cluster for genes involved in cell cycle progression and regulation, and found that *Fpr2* deletion significantly decreased the mRNA levels of genes involved in the G1 to S phase transition (*Ccnd1*, *Ccnd2*, *Ccne1*, *Ccne2*, and *E2f2*) (Supplementary Fig. S5a−d and f), S to G2/M phase transition (*Ccna2*) (Supplementary Fig. S5e), and DNA replication (*Dbf4*, *Mcm4*, *Mcm5*, *Mcm6*, *Orc6*, *Cdc45*, *Gins1*, and *Gins2*) (Supplementary Fig. S5g−n), as well as cell proliferation markers (*Mki67* and *Pcna*) (Supplementary Fig. S5o and p). The altered expression of the above genes was validated in the small intestinal crypts of *Fpr2^VKO^* mice (Supplementary Fig. S5q−s). These results support that deletion of intestinal epithelial *Fpr2* impairs ISC proliferation by inhibiting cell cycle progression from G1 to S and G2/M phase transitions.

**Figure 5:**
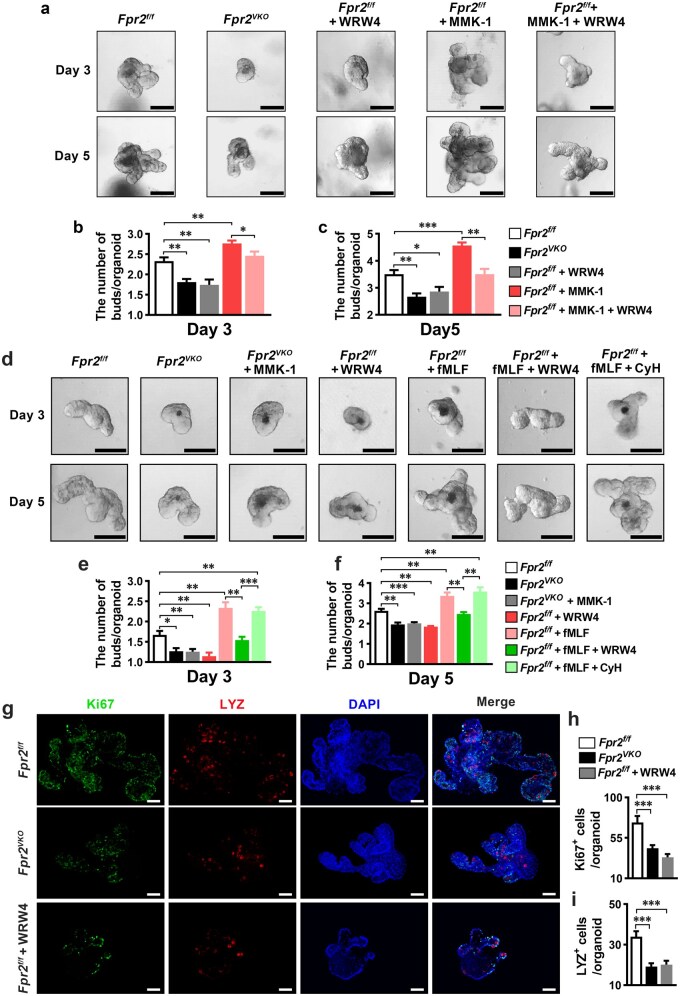
Activation of intestinal epithelial FPR2 promotes the growth and budding of organoids derived from small intestinal crypts. Crypts were isolated from the ileum of *Fpr2^f/f^* and *Fpr2^VKO^* mice, followed by culturing with or without the following: 1 µmol/L MMK-1, 10 µmol/L fMLF, 1 µmol/L MMK-1, and 10 µmol/L WRW4, or 10 µmol/L fMLF and 1 µmol/L CyH. (a–f) Representative images of ileal organoids cultured for 3 and 5 days (a and d), and quantification of the number of buds per organoid (b, c, e, and f). Scale bar, 100 μm. *n *= 5/group. More than 130 organoids were counted in each group. (g–i) Representative images of immunofluorescence staining for Ki67 (green) and LYZ (red) in ileal organoids cultured for 5 days. Cell nuclei were stained with DAPI. Scale bar, 50 μm. CyH, cyclosporine H. Each group of organoids was cultured from the ileal crypts of three mice, and more than 30 organoids were counted in each group. Data are presented as mean ± SEM. ^*^P < 0.05; ^**^P < 0.01; ^***^P < 0.001, by one-way ANOVA.

The effect of FPR2 on ISC proliferation and differentiation was further investigated in intestinal organoids derived from small intestinal crypts of *Fpr2^f/f^* and *Fpr2^VKO^* mice. Organoids derived from *Fpr2^VKO^* mice exhibited fewer buds 3 and 5 days after culture compared to those derived from *Fpr2^f/f^* mice. Treatment of small intestinal crypts from *Fpr2^f/f^* mice with the FPR2 antagonist WRW4 reduced the number of organoid buds. In contrast, the FPR2 agonist MMK-1 had the opposite effect. Co-treatment of crypts from *Fpr2^f/f^* mice with MMK-1 and WRW4 reversed the pro-budding effect of MMK-1 (Fig. 5a−c). MMK-1 did not significantly affect the growth and budding of small intestinal organoids derived from *Fpr2^VKO^* mice (Fig. 5d−f). These results indicate that FPR2 activation plays a cri­tical role in organoid growth and budding. We also found that N-formyl-methionyl-leucyl-phenylalanine (fMLF), an FPR1/2 agonist produced by the gut microbiota, significantly promoted the growth and budding of organoids derived from *Fpr2^f/f^* mice. This effect of fMLF was reversed by WRW4, but not by the FPR1 anta­gonist cyclosporine H (Fig. 5d−f), indicating that fMLF promotes organoid growth and budding via FPR2. Immunofluorescence staining showed that both Ki67^+^ proliferative cells and LYZ^+^ Paneth cells were significantly reduced in organoids derived from *Fpr2^VKO^* mice compared to those derived from *Fpr2^f/f^* mice. Treatment of small intestinal crypts derived from *Fpr2^f/f^* mice with WRW4 resulted in a significant decrease in Ki67^+^ cells and LYZ^+^ Paneth cells in the organoids (Fig. 5g−i). Taken together, these results indicate that activation of FPR2 in ISCs promotes organoid growth and budding by stimulating ISC proliferation and differentiation.

### FPR2 promotes ISC proliferation and differentiation by activating the Wnt, Notch, and Hippo signaling pathways via protein kinase C (PKC)−extracellular signal-regulated kinase (ERK)

**Figure 6:**
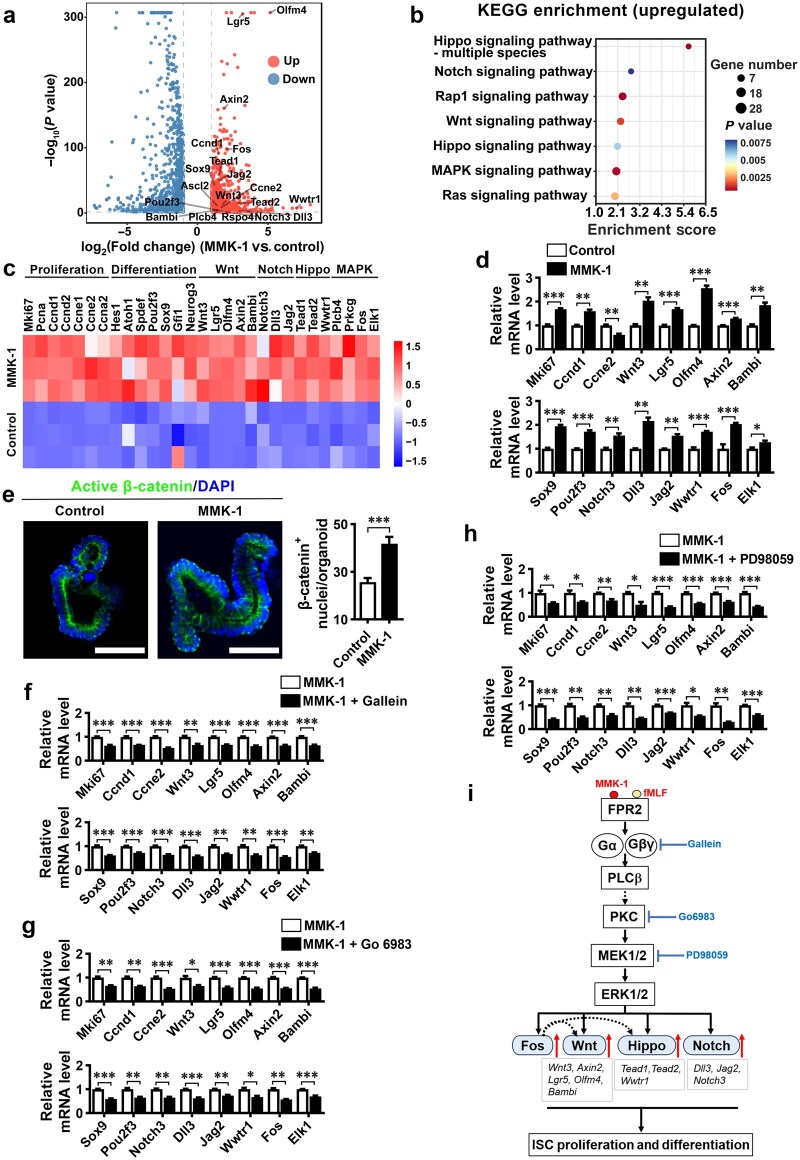
Activation of FPR2 promotes the proliferation and differentiation of ISCs by activating the Wnt, Hippo, and Notch signaling pathways via PKC-ERK. Ileal organoids derived from mice were treated with 1 μmol/L MMK-1 and examined for gene expression via RNA-seq. (a) Volcano plot showing DEGs in MMK-1-treated versus control organoids. *n *= 3/group. (b) KEGG pathway enrichment analysis of genes upregulated by MMK-1. (c) Heatmaps showing MMK-1-regulated genes involved in cell proliferation and ISC differentiation, as well as genes in the Wnt, Notch, Hippo, and MAPK signaling pathways, as detected via RNA-seq. (d) RT-qPCR analysis to verify the expression of MMK-1-regulated genes in organoids. (e) Representative images of immunofluorescence staining for active β-catenin (green) in MMK-1-treated organoids, along with a quantification of β-catenin-positive nuclei per organoid. Scale bar, 50 μm. (f−h) Expression analysis of MMK-1-induced genes treated with the Gβγ inhibitor Gallein, the PKC inhibitor Go6983, or the MEK inhibitor PD98059. The impact of Gallein (10 μmol/L), Go6983 (1 μmol/L), and PD98059 (10 μmol/L) on the aforementioned gene expression in organoids was examined via RT-qPCR analysis. (i) A schematic diagram illustrating the signaling pathways by which FPR2 activation regulates ISC proliferation and differentiation. *n *= 3/group. Each sample contains organoids derived from three mice. Data are presented as mean ± SEM. ^*^P < 0.05; ^**^P < 0.01; ^***^P < 0.001, by unpaired two-tailed Student’s *t*-test.

The molecular mechanisms underlying the regulation of ISC proliferation and differentiation by FPR2 was investigated by performing RNA-seq to analyze gene expression in mouse ileal organoids treated with MMK-1. A total of 964 genes were upregulated and 997 genes were downregulated by MMK-1 treatment (Fig. 6a). Kyoto Encyclopedia of Genes and Genomes (KEGG) enrichment analysis of the upregulated genes revealed significant enrichment in the MAPK, Wnt, Notch, and Hippo signaling pathways (Fig. 6b). These pathways are known to regulate ISC proliferation and TA cell differentiation [35–38]. Genes associated with cell proliferation, including cell proliferation marker genes (*Mki67* and *Pcna*)*,* cyclin genes involved in the progression of cell cycle from G1 to S and G2 phases (*Ccnd1/2*, *Ccne1/2*, and *Ccna2*), and transcription factors involved in ISC differentiation (*Atoh1*, *Spdef*, *Pou2f3*, *Sox9*, and *Gfi1*), were significantly upregulated in MMK-1-treated organoids. Wnt signaling pathway genes, including *Wnt3* and Wnt target genes (*Lgr5*, *Olfm4*, *Axin2*, and *Bambi*), as well as genes in Notch (*Notch3*, *Dll3*, and *Jag2*), Hippo (*Tead1/2* and *Wwtr1*), and MAPK (*Plcb4*, *Prkcg*, *Fos*, and *Elk1*) pathways, were markedly upregulated in MMK-1-treated organoids (Fig. 6c). RT-qPCR analysis confirmed the upregulation of these genes induced by MMK-1 treatment in organoids (Fig. 6d). scRNA-seq of ileal crypts revealed that *Wnt3* was expressed in various IEC types, with the highest level in Paneth cells (Supplementary Fig. S6a−e). Wnt target genes (*Axin2*, *Bambi*, and *Ccnd1*) were primarily expressed in ISCs and TA cells (Supplementary Fig. S6f). Intestinal epithelial *Fpr2* deficiency significantly decreased *Wnt3* expression in Paneth cells, and *Ccnd1* expression in both ISCs and TA cells (Supplementary Fig. S6b, S6g, and S6h). Since cyclin D1, which is encoded by *Ccnd1*, plays an important role in the G1/S transition of the cell cycle, these results suggest that FPR2 induces *Wnt3* expression in Paneth cells. Wnt3 then promotes the prolife­ration of ISCs and TA cells. Immunofluorescence staining of active β-catenin in organoids revealed that MMK-1 treatment increased β-catenin nuclear localization (Fig. 6e), demonstrating that FPR2 activation leads to activation of the Wnt signaling pathway. scRNA-seq of ileal crypts revealed that Notch pathway genes were primarily expressed in Paneth cells, tuft cells, and EE cells (Supplementary Fig. S7a), and Hippo pathway genes were mainly expressed in EE and Tuft cells (Supplementary Fig. S7e). Intestinal *Fpr2* deficiency decreased Notch ligands, including *Jag2* in Paneth and EE cells (primarily EE cells), and *Dll3* in EE and tuft cells. It also decreased *Notch3* expression in EE and tuft cells (Supplementary Fig. S7b−d). Intestinal epithelial *Fpr2* deficiency also decreased the expression of Hippo pathway effector genes, including *Tead1* in EE cells, *Tead2* in tuft cells, and *Wwtr1* in both EE and tuft cells (Supplementary Fig. S7f and g). Together with the upregulation of these genes in intestinal organoids by MMK-1 treatment, these results suggest that FPR2 activation upregulates Notch and Hippo pathway genes in EE and tuft cells.

FPR2 is a G protein-coupled receptor whose activation triggers the activation of ERK through PKC [39]. Since ERK has been reported to modulate the Wnt and Hippo pathways [38, 40–42], and the Notch pathway is activated by MMK-1 via FPR2 (Supplementary Fig. S8a and b), we examined whether the G protein, PKC, ERK, and the Wnt, Hippo, and Notch signaling pathways are involved in MMK-1-induced ISC proliferation and differentiation. Treatment of ileal organoids with the FPR2 antagonist WRW4, the Gβγ inhibitor Gallein, the PKC inhibitor Go6983, or the mitogen-activated protein kinase kinase (MEK) inhibitor PD98059 significantly reduced MMK-1-induced expression of key genes in the MAPK, Wnt, Hippo, and Notch signaling pathways, and genes involved in cell proliferation and ISC differentiation (Fig. 6f−h; Supplementary Fig. S8a and b). These results suggest that FPR2 activation promotes ISC proliferation and differentiation via PKC-MEK-dependent activation of the Wnt, Notch, and Hippo pathways (Fig. 6i). We also found that, like MMK-1, bacterial peptide fMLF upregulated key genes in the MAPK, Wnt, Hippo, and Notch signaling pathways, and genes involved in cell proliferation and ISC differentiation in the ileal organoids. The inductive effect of fMLF was blocked by WRW4 (Supplementary Fig. S8c and d). WRW4 reduced the expression of these genes in organoids, and IEC *Fpr2* deficiency resulted in decreased expression of these genes in organoids and small intestinal crypts of mice (Supplementary Fig. S8e−g). These results suggest that under physiological conditions, FPR2 activation by agonists produced by host and gut microbiota promotes ISC proliferation and differentiation via PKC-ERK-dependent Wnt, Notch, and Hippo signaling pathways (Fig. 6i). FBJ murine osteosarcoma viral oncogene homologue (FOS) is a transcription factor with binding sites in the promoter regions of genes in the Wnt and Hippo signaling pathways (Supplementary Table S1). *Fos* was upregulated by MMK-1 through MEK (Fig. 6h), and by fMLF (Supplementary Fig. S8c). It was downregulated by WRW4 or *Fpr2* knockout (Supplementary Fig. S8e−g). Thus, the upregulation of Fos by MMK-1 may stimulate the expression of genes in the Wnt and Hippo signaling pathways, contributing to ISC proliferation and differentiation (Fig. 6i).

We further examined the effect of FPR2 activation on ISC proliferation and differentiation *in vivo*. Administration of MMK-1 did not affect body weight of the mice (Supplementary Fig. S9a), but led to a significant increase in the villus height and crypt depth of the small intestine after 5 and 7 days (Supplementary Fig. S9b−d). Immunohistochemical staining of Ki67 and OLFM4 showed a significant increase in the numbers of Ki67^+^ proliferative cells and OLFM4^+^ ISCs in small intestinal crypts on days 5 and 7 after MMK-1 treatment (Supplementary Fig. S9e and f). This increase was accompanied by the upregulation of key genes in the MAPK, Wnt, Hippo, and Notch signaling pathways, as well as genes involved in cell proliferation and ISC differentiation (Supplementary Fig. S9g and h). Taken together, these results suggest that FPR2 activation promotes the proliferation and differentiation of ISCs and TA cells via the MAPK, Wnt, Hippo, and Notch signaling pathways.

### Intestinal epithelial *Fpr2* deficiency impairs intestinal epithelium regeneration after injury by reducing ISC regeneration and TA cell proliferation in mice

**Figure 7:**
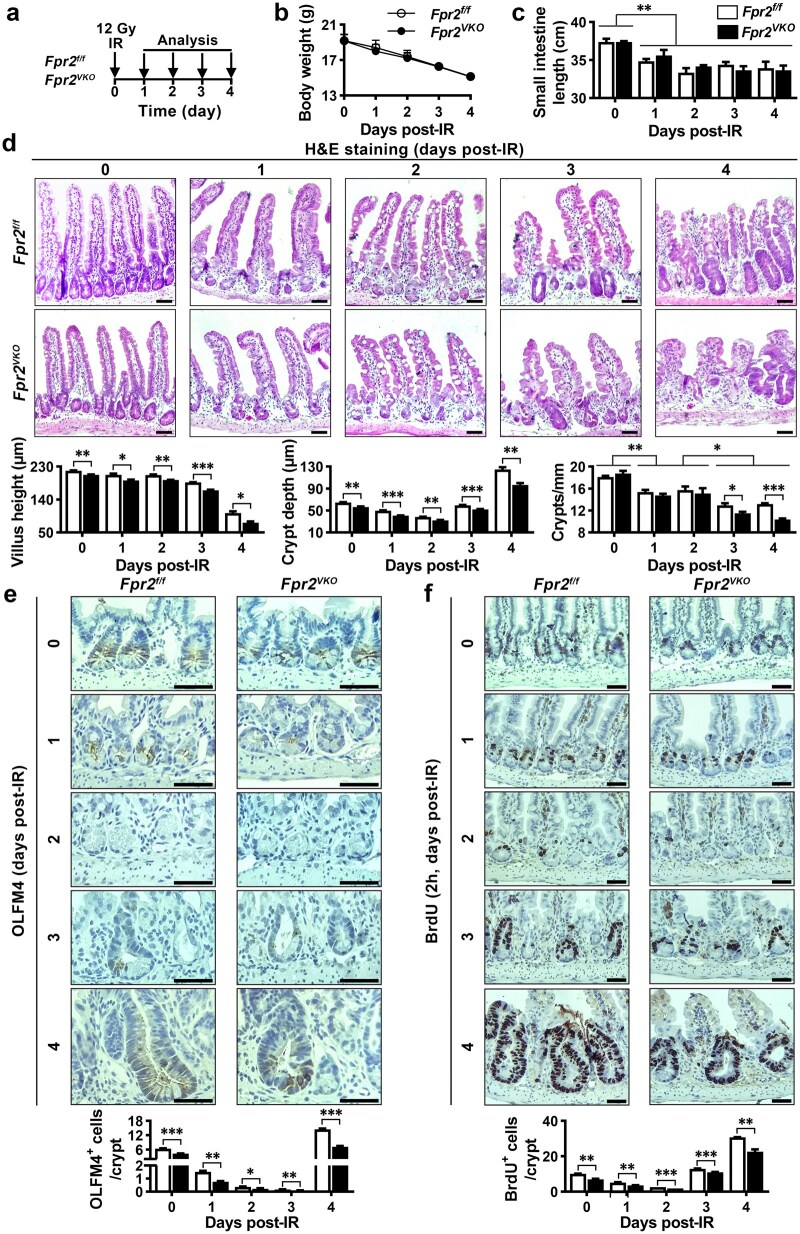
Intestinal epithelial *Fpr2* deficiency in mice impairs small intestinal epithelium regeneration after irradiation-induced injury. (a) Schematic diagram of the experiments. *Fpr2^f/f^* and *Fpr2^VKO^* mice were exposed to 12 Gy X-rays and small intestines were harvested for analysis at different time points after IR. (b and c) Body weight (b) and small intestine length (c) of *Fpr2^f/f^* and *Fpr2^VKO^* mice after IR. (d) Representative H&E staining images of small intestine sections from *Fpr2^f/f^* and *Fpr2^VKO^* mice after IR, with quantification of villus height, crypt depth, and number of crypts per millimeter. (e) Representative images of immunohistochemical staining for OLFM4 in small intestine sections from *Fpr2^f/f^* and *Fpr2^VKO^* mice after IR, with quantification of OLFM4^+^ stem cells at the indicated time after IR. (f) Representative images of BrdU immunohistochemical staining of small intestine sections after intraperitoneal injection of BrdU for 2 h at the indicated time after IR in *Fpr2^f/f^* and *Fpr2^VKO^* mice, with quantification of positive cells. *n *= 3 mice/group on days 0, 1, 2 and 4; *n *= 5 mice/group on day 3 after IR. Scale bar, 50 μm. Data are presented as mean ± SEM. ^*^P < 0.05; ^**^P < 0.01; ^***^P < 0.001, by unpaired two-tailed Student’s *t*-test.

We investigated whether FPR2 contributes to intestinal epithelium regeneration after injury. After 12-Gy IR (Fig. 7a), both *Fpr2^f/f^* and *Fpr2^VKO^* mice showed progressive weight loss (Fig. 7b) and shortening of the small intestine (Fig. 7c). H&E staining of small intestine sections showed a progressive decrease in villus height and crypt depth, followed by a gradual recovery of crypt depth after IR. Compared to *Fpr2^f/f^* mice, *Fpr2^VKO^* mice had significantly shorter villi and shallower crypts at all time points examined, along with a marked reduction in the number of crypts per mm at days 3 and 4 after IR (Fig. 7d). Immunohistochemical staining revealed a progressive decrease in OLFM4^+^ ISCs in the small intestinal crypts of *Fpr2^f/f^* mice after IR, with nearly complete absence by day 3, followed by a significant increase on day 4. However, *Fpr2^VKO^* mice exhibited a more significant decrease and smaller increase in OLFM4^+^ ISCs after IR (Fig. 7e). A BrdU incorporation study in *Fpr2^f/f^* mice showed that the number of BrdU^+^ cells in the crypts decreased progressively after IR, followed by a significant increase starting on day 3. These alterations are consistent with the changes in crypt depth after IR. All cells in the crypts were BrdU positive on day 4 after IR, indicating the proliferation of ISCs and TA cells. However, *Fpr2^VKO^* mice had fewer BrdU^+^ cells in the crypts than *Fpr2^f/f^* mice after IR (Fig. 7f). These results demonstrate that intestinal epithelial *Fpr2* deficiency delays intestinal epithelium repair after IR-induced injury by impairing the regeneration of ISCs, and the proliferation of ISCs and TA cells. Since *Fpr2^f/f^* and *Fpr2^VKO^* mice exposed to 12 Gy of X-ray died after 5 to 6 days, the radiation dose was reduced to 10 Gy. *Fpr2^VKO^* mice exposed to 10 Gy had lower body weight and survival rate than *Fpr2^f/f^* mice. Villus height and crypt depth in the small intestine fully recovered by day 7 post-IR in *Fpr2^f/f^* mice, but only partially recovered in *Fpr2^VKO^* mice (Supplementary Fig. S10). These results confirmed that intestinal epithelial *Fpr2* deficiency impairs regeneration of the intestinal epithelium following IR-induced injury.

**Figure 8:**
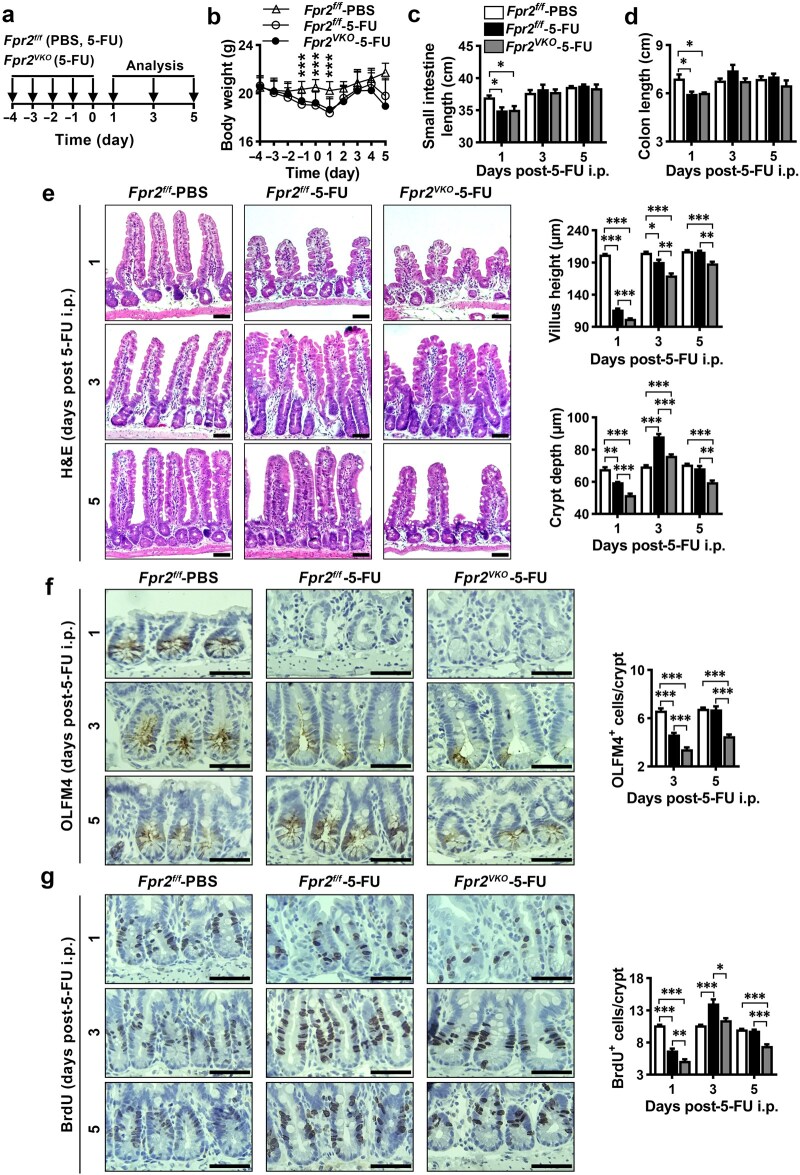
Intestinal epithelial *Fpr2* deficiency in mice impairs small intestinal epithelium regeneration after 5-FU-induced injury. (a) Schematic diagram of the experiments. *Fpr2^f/f^* and *Fpr2^VKO^* mice were injected intraperitoneally with 5-FU (50 mg/kg body weight/day) or equal volume of PBS, and small intestines were harvested at different time points for analysis. (b–d) Body weight (b), small intestine length (c), and colon length (d) of *Fpr2^f/f^* and *Fpr2^VKO^* mice during and after 5-FU or PBS treatment. (e) Representative H&E staining images of small intestine sections from *Fpr2^f/f^* and *Fpr2^VKO^* mice after 5-FU or PBS treatment, with quantification of villus height and crypt depth. (f) Representative OLFM4 immunohistochemical staining images of small intestine sections from 5-FU- or PBS-treated *Fpr2^f/f^* and *Fpr2^VKO^* mice, with quantification of OLFM4^+^ stem cells. (g) Representative BrdU immunohistochemical staining images of small intestine sections after intraperitoneal injection of BrdU for 2 h at the indicated time points after 5-FU or PBS treatment in *Fpr2^f/f^* and *Fpr2^VKO^* mice, with quantification of BrdU^+^ cells. *n *= 6 mice/group. Scale bar, 50 μm. Data are presented as mean ± SEM. ^*^P < 0.05; ^**^P < 0.01; ^***^P < 0.001, by two-way ANOVA.

The involvement of FPR2 in small intestinal epithelial repair was further investigated in 5-FU-treated mice (Fig. 8a). Compared with phosphate-buffered saline (PBS)-treated *Fpr2^f/f^* mice, body weight of *Fpr2^f/f^* and *Fpr2^VKO^* mice decreased continuously during 5-FU administration (Fig. 8b). Body weight and the length of the small intestine and colon in *Fpr2^f/f^* and *Fpr2^VKO^* mice were still lower than that of PBS-treated *Fpr2^f/f^* mice on day 1 after 5-FU withdrawal but recovered thereafter (Fig. 8b−d). H&E staining of small intestine sections showed that compared with PBS-treated *Fpr2^f/f^* mice, villus height and intestinal crypt depth in *Fpr2^f/f^* mice were smaller on day 1 after 5-FU withdrawal and recovered on day 5. After 5-FU withdrawal, villus height and intestinal crypt depth in *Fpr2^VKO^* mice showed a pattern of change similar to that of *Fpr2^f/f^* mice, but significantly lower than that of *Fpr2^f/f^* mice (Fig. 8e). These results suggest that *Fpr2* deletion in the intestinal epithelium exacerbated 5-FU-induced villus and crypt injury, and delayed recovery. Immunohistochemical staining showed that after 5-FU withdrawal, OLFM4^+^ ISCs were almost completely lost on day 1 and reappeared on day 3 in both *Fpr2^f/f^* and *Fpr2^VKO^* mice, and the number of OLFM4^+^ ISCs recovered on day 5 in *Fpr2^f/f^* mice but not in *Fpr2^VKO^* mice (Fig. 8f). BrdU incorporation study showed that BrdU^+^ cells in small intestinal crypts of *Fpr2^f/f^* mice were significantly reduced on day 1, increased on day 3, and returned to normal on day 5 after 5-FU withdrawal, which was consistent with the change in crypt depth. However, BrdU^+^ cells in crypts of *Fpr2^VKO^* mice were always significantly lower than those of *Fpr2^f/f^* mice during this period (Fig. 8g). These results indicate that *Fpr2* deletion delays intestinal epithelium repair after 5-FU-induced injury by impairing ISC regeneration and TA cell proliferation.

### Treatment of mice with MMK-1 promotes intestinal epithelium regeneration from irradiation-induced injury

**Figure 9:**
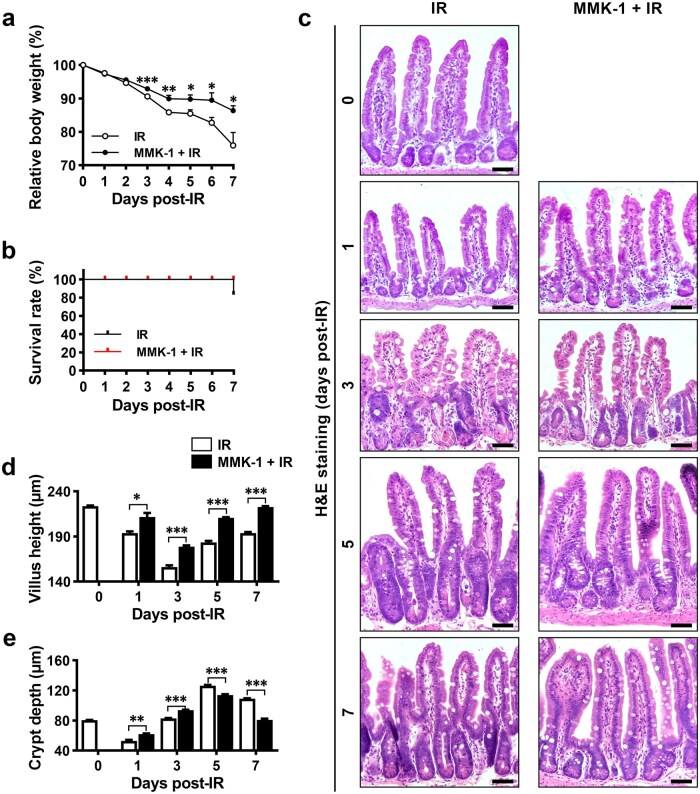
Activation of FPR2 promotes intestinal epithelium regeneration after irradiation in mice. Mice were administered either MMK-1 (10 mg/kg body weight/day, intraperitoneally) or an equal volume of PBS on the day of 10-Gy IR and for the subsequent 5 days. The small intestines were harvested at different time points after IR to examine histology. (a and b) Body weight (a) and survival rates (b) of mice after IR. (c–e) Representative H&E-stained images of small intestinal sections (c), and quantitative analyses of villus height (d) and crypt depth (e), at the indicated time points after IR. *n *= 6 mice/group on days 0, 3, 5, and 7 post-IR; *n *= 3 mice/group on day 1 post-IR. Scale bar, 50 μm. Data are presented as mean ± SEM. ^*^P < 0.05; ^**^P < 0.01; ^***^P < 0.001, by unpaired two-tailed Student’s *t* test.

Because intestinal epithelial *Fpr2* deficiency impairs the repair of intestinal epithelium after injury, we investigated whether FPR2 activation could promote regeneration of the small intestinal epithelium after IR. We found that administrating MMK-1 to mice significantly reduced weight loss and increased survival rate after IR (Fig. 9a and b). Villus height decreased significantly at 1 and 3 days post-IR. It increased after 5 days, but did not fully recover by day 7. Crypt depth decreased significantly 1 day after IR, increased 3 days after IR, peaked 5 days after IR, and decreased 7 days after IR; however, it remained larger than that of control mice. The patterns of alteration in villus height and crypt depth after IR were similar in MMK-1- and vehicle-treated mice. However, MMK-1 treatment resulted in a smaller decrease in villus height at 1 and 3 days post-IR and full recovery of villus height and crypt depth by day 7 post-IR (Fig. 9c–e). These results demonstrate that FPR2 activation promotes intestinal epithelium regeneration after IR and indicate that FPR2 is a therapeutic target for treating intestinal epithelial injury induced by radiotherapy.

## Discussion

In this study, we investigated the role of intestinal epithelial FPR2 in intestinal epithelium homeostasis and regeneration, and the associated mechanisms. We found that deletion of *Fpr2* in either IECs or ISCs in mice reduced multiple types of IECs in the small intestine and colon, and reduced villus height and crypt depth. Intestinal epithelial *Fpr2* deficiency delayed intestinal epithelium regeneration after IR and 5-FU-induced intestinal injury by reducing ISC regeneration and TA cell proliferation. Administering an FPR2 agonist to mice significantly increased survival rate and accelerated intestinal epithelium regeneration after irradiation. These results indicate that FPR2 plays a critical role in maintaining intestinal epithelium homeostasis and regeneration after injury. Mechanistic studies revealed that FPR2 activation promotes ISC and TA cell proliferation and differentiation by activating the Wnt, Hippo, and Notch signaling pathways via PKC-ERK.

We found that in *Fpr2^VKO^* mice, intestinal villus length and crypt depth were significantly decreased, accompanied by the reduction of enterocytes, goblet cells, Paneth cells, OLFM4^+^ ISCs, and proli­ferative crypt cells in the small intestine and colon, compared to *Fpr2^f/f^* mice. *Lgr5-Fpr2^KO^* mice showed intestinal epithelium and IEC phenotypes that were similar to those of *Fpr2^VKO^* mice. Since ISCs and TA cells are the only proliferative cells in intestinal crypts, and TA cells are derived from ISCs and differentiate into different types of IECs, the above results indicate that epithelial FPR2 plays a ­critical role in maintaining intestinal epithelium homeostasis by regulating ISC and TA cell proliferation. Consistent with this, our previous collaborative research showed that systemic *Fpr2* ­knockout in mice results in shallower colonic crypts and reduced crypt cell proliferation [20].

To explore the mechanisms involved in the regulation of ISC proliferation by intestinal epithelial FPR2, we performed scRNA-seq of small intestinal crypt epithelial cells from *Fpr2^f/f^* and *Fpr2^VKO^* mice, as well as bulk RNA-seq of small intestinal organoids treated with the FPR2 agonist MMK-1. The scRNA-seq results revealed decreased numbers of enterocytes, TA cells, goblet cells, and Paneth cells in *Fpr2^VKO^* mice. This finding is consistent with reduced expression of marker genes for these cell types in the small intestine, as determined by PAS staining, immunostaining, and RT-qPCR. Analysis of the scRNA-seq data revealed a significant increase in ICs in the small intestinal crypts of *Fpr2^VKO^* mice. These results suggest that *Fpr2* deficiency reduces mature IECs in the small intestinal epithelium by primarily impairing the terminal differentiation of TA cells. A thorough examination of the scRNA-seq-derived transcriptional profile of ICs in *Fpr2^f/f^* and *Fpr2^VKO^* mice indicates that *Fpr2* deficiency inhibits IC proliferation and differentiation, potentially through the downregulation of genes associated with protein synthesis, cell proliferation, and differentiation. Analysis of the ISC transcriptional profile revealed that *Fpr2* deletion downregulated genes involved in the G1/S transition of cell cycle, S-phase DNA replication, and the G2/M transition. Among the ISC subclusters, *Fpr2* deficiency increased the Lgr5 stem cell subcluster consisting of cells in the G1 phase. These results indicate that *Fpr2* deficiency in the intestinal epithelium inhibits ISC proliferation by suppressing the progression of cell cycle from G1 to S and G2/M phases. This conclusion is supported by flow cytometry analysis, which revealed that *Fpr2* deficiency in ISCs inhibited ISC proliferation by blocking cell cycle progression from G1 to S and G2/M phases. Bulk RNA-seq analysis of small intestinal organoids treated with the FPR2 agonist MMK-1 revealed that FPR2 activation significantly increased the expression of genes associated with cell proliferation and ISC differentiation, as well as the expression of genes in Wnt, Notch, and Hippo signaling pathways, via activation of the FPR2−PKC−ERK pathway. The bacterial peptide fMLF has a similar effect to MMK-1 in regulating gene expression and organoid growth and budding. In mice, administration of MMK-1 upregulated these genes, promoted crypt cell proliferation, and increased ISCs in the small intestine. It also increased the villus height and crypt depth of the small intestine. Conversely, we found that these genes were downregulated in the small intestinal organoids and small intestines derived from *Fpr2^VKO^* mice. Analysis of scRNA-seq data revealed that intestinal *Fpr2* deficiency significantly reduced the expression of Wnt3 in Paneth cells and its target genes in ISCs and TA cells, and reduced the expression of Notch ligands and Hippo pathway effector genes in EE and tuft cells. Wnt signaling is crucial for ISC self-renewal and TA cell proliferation, as well as the initiation of differentiation towards secretory IECs and the maturation of Paneth cells [35, 43, 44]. Notch signaling is required for ISC self-renewal, while elevated Notch activity promotes enterocyte differentiation [36]. Therefore, our findings suggest that FPR2 activation promotes ISC and TA cell proliferation by upregulating Wnt3 in Paneth cells and Notch ligands in EE and tuft cells. Additionally, upregulation of Wnt3 contributes to the differentiation initiation of secretory IECs and the maturation of Paneth cells, while upregulation of Notch ligands contributes to the differentiation of absorptive enterocytes. The Hippo−YAP/TAZ pathway contributes to intestinal self-renewal and regeneration after injury by promoting ISC and TA cell proliferation [37]. Activation of the YAP/TAZ pathway stimulates ISC proliferation by binding to the transcription factor transcriptional enhanced associate domains (TEADs) and promotes the differentiation of goblet cells by cooperating with the transcription factor Kruppel-like factor 4 (KLF4) [45]. G protein-coupled receptors stimulate cell proliferation by activating the YAP/TAZ pathway [46]. We found that MMK-1 increased the expression of *Wwtr1* (TAZ), *Tead1*, and *Tead2*, and stimulated small intestinal organoid growth and budding via FPR2. However, analysis of scRNA-seq data of small intestinal crypt ECs revealed that intestinal epithelial *Fpr2* deficiency had no significant effect on the expression of these genes in ISCs and TA cells. These results suggest that FPR2 activation promotes ISC proliferation and differentiation through the YAP/TAZ pathway; however, this pathway is not involved in FPR2-mediated regulation of ISC proliferation and differentiation under physiological conditions.

The ligands that activate intestinal epithelial FPR2 under phy­siological conditions are not clear. The length of colonic crypts in systemic *Fpr2* knockout (*Fpr2^KO^*) mice was not different from that of WT mice within 30 days of birth, but decreased significantly thereafter [47], suggesting that exogenous factors contribute to colonic epithelium homeostasis via FPR2. Gut microbiota plays an important role in intestinal epithelium homeostasis. Oral ingestion of gut microbiota in germ-free (GF) mice or *Lactobacillus rhamnosus* GG in GF rats increases mitotic cells in small intestinal crypts, the depth of crypts, and the cells in the intestinal villus and crypts [48, 49]. The following evidence supports our hypothesis that intestinal epithelial FPR2 is activated by ligands produced by gut bacteria and IECs under physiological conditions, thereby contributing to intestinal epithelium homeostasis. (ⅰ) Gut microbiota and IECs produce FPR2 agonists, such as bacteria-derived formyl peptides and crypt cell-secreted cathelin-related antimicrobial peptide (CRAMP) [16, 50, 51]. (ⅱ) Intestinal epithelium-specific *Cramp* gene knockout mice have shortened colonic crypts and reduced numbers of goblet cells and proliferative cells in the crypts [50], similar to *Fpr2^KO^* and *Fpr2^VKO^* mice. Our present study revealed that intestinal epithelial *Fpr2* deficiency led to reduced expression of *Cramp* in the intestine (Supplementary Fig. S11). (ⅲ) Deficiency of FPR2 or CRAMP in the intestinal epithelium results in intestinal dysbiosis [47, 50, 52]. CRAMP contributes to colon homeostasis by maintaining the balance of gut microbiota [52]. (ⅳ) Intestinal commensal *Escherichia coli* stimulates *Fpr2* expression in colonic epithelial cells, and *E. coli* products, fMLF, and CRAMP induce IEC proliferation via FPR2 [20, 47, 50]. The present study demonstrated that fMLF upregulates genes associated with cell proliferation and ISC differentiation, and promotes intestinal organoid growth and budding via FPR2.

The intestine is a highly sensitive organ that is injured during radiotherapy and chemotherapy for malignant tumors. We found that intestinal epithelial *Fpr2* deletion delayed small intestinal epithelium regeneration after IR and 5-FU-induced intestinal injury by reducing the regeneration and proliferation of ISCs and the proliferation of TA cells. Recent studies have shown that proliferation of reserve stem cells and dedifferentiation of both secretory/absorptive progenitors and Paneth cells into ISCs contribute to intestinal epithelial regeneration after injury [53]. Whether *Fpr2* deficiency delays intestinal epithelium repair by impairing proli­feration and/or dedifferentiation of these cells requires further investigation. Kim *et al.* reported that the FPR1/2 agonist WKYMVm ameliorates DSS-induced colitis in mice and induces colonic epithelial cell proliferation via FPR2 [54]. Chen *et al.* reported that administration of commensal *E. coli* to GF mice alleviates DSS-induced colon injury by upregulating FPR2 expression in colonic epithelial cells and promoting crypt cell proliferation [47]. These results support that FPR2 is essential for intestinal epithelium regeneration after injury. It has been reported that mitochondrial formyl peptides released from injured tissues are FPR2 agonists [16, 55, 56]. Therefore, mitochondria-derived FPR2 formyl peptides after X-ray and 5-FU-induced intestinal injury may activate FPR2 to stimulate ISC proliferation and differentiation. The present study demonstrated that MMK-1 administration significantly mitigated body weight loss and mortality, and facilitated the regeneration of intestinal villi and crypt after IR-induced injury. These results suggest that FPR2 may serve as a viable therapeutic target for the treatment of radiotherapy-induced intestinal injury.

In summary, the present study identified intestinal epithelial FPR2 as a key regulator of intestinal epithelium homeostasis under physiological conditions and during regeneration following injury. FPR2 activation promotes ISC and TA cell proliferation and differentiation by activating the Wnt, Notch, and Hippo signaling pathways via PKC-ERK. Wnt and Notch signaling pathways are involved in FPR2-mediated regulation of ISC proliferation and differentiation under physiological conditions. FPR2 accelerates regeneration of the intestinal epithelium following radiation and chemotherapy-induced injury by stimulating the proliferation and differentiation of ISCs and TA cells. Therefore, FPR2 is a promising therapeutic target for treating intestinal injury induced by chemotherapy and radiotherapy.

## Limitations of the study

This study identified intestinal epithelial FPR2 as a key regulator of intestinal epithelial homeostasis and regeneration in mice, and revealed that FPR2 promotes ISC and TA cell proliferation and differentiation through the PKC-ERK signaling pathway-mediated upregulation of Wnt3 expression in Paneth cells and Notch ligand expression in EE and tuft cells under physiological conditions. However, the endogenous ligands that activate epithelial FPR2 under physiological conditions and during intestinal epithelial regeneration following injury require further investigation. Additionally, it is unclear whether intestinal epithelial FPR2 functions similarly in humans.

## Materials and methods

### Mice and treatments

*Fpr2^flox/flox^* (*Fpr2^f/f^)* mice on the C57BL/6J background were generated as described previously [57]. *Lgr5-EGFP-IRES-creERT2 (Lgr5-GFP)* mice were obtained from Y.A.Z*. Fpr2^f/f^* mice were crossed with Villin-Cre mice obtained from the Model Animal Research Center of Nanjing University (Nanjing, China) to generate *Fpr2^VKO^* mice, or crossed with *Lgr5-GFP* mice to generate *Fpr2^f/f^; Lgr5-GFP* mice. *Fpr2^f/f^*; *Lgr5-GFP* mice were injected intraperitoneally with tamoxifen (75 mg/kg body weight/day) for 5 days to induce *Fpr2* deletion in ISCs or received the same amount of corn oil as control. To detect IEC proliferation, mice were injected intraperitoneally with BrdU (50 mg/kg body weight), and euthanized after 2 h. The intestines were harvested for immunofluorescence staining of BrdU. To induce intestinal injury, mice were irradiated with a cabinet X-ray irradiator (CIX3, Xstrahl, Surrey, UK) at a dose of 10 or 12 Gy, or were injected intraperitoneally with 5-FU (50 mg/kg body weight/day) for 5 consecutive days. To evaluate the impact of FPR2 activation on intestinal injury repair, mice received intraperitoneal injections of either MMK-1 (10 mg/kg body weight/day) or an equal volume of PBS on the day of 10-Gy IR and for 5 subsequent days. The small intestines were harvested at various timepoints post-IR for histology and gene expression analysis. Mice that received the same dose of MMK-1 and an equal volume of PBS were used to examine the contribution of FPR2 to intestinal epithelium homeostasis. Sex- and age-matched mice aged 8–12 weeks were used for all experiments and randomly allocated to control and treatment groups. Mice were maintained in specific pathogen-free animal facility with *ad libitum* access to food and water. All animal experimental protocols were approved by the Institutional Animal Care and Use Committee of the Shanghai Institute of Nutrition and Health, Chinese Academy of Sciences.

#### H&E staining, TUNEL assay, and immunostaining

Mouse intestinal samples were fixed in 4% paraformaldehyde, embedded in paraffin, and sectioned at 5-μm thickness. Sections were stained with H&E. Goblet cells were stained with a Periodic Acid-Schiff Staining Kit (Beyotime Biotechnology, Shanghai, China) according to the manufacturer’s instructions. Apoptotic cells were determined using a One Step TUNEL Apoptosis Assay Kit (Beyotime Biotechnology, Shanghai, China) according to the manufacturer’s protocol. Cell nuclei were stained with 4’-6-diamidino-2-phenylindole (DAPI). Images were captured using a Revolve inverted integrated fluorescence microscope (RVL-100-G, ECHO, San Diego, CA, USA) or a confocal laser scanning microscope (FV1200, Olympus, Tokyo, Japan). Quantification of villus height, crypt depth, PAS-positive cells, and TUNEL-positive cells was performed using Image-Pro Plus 6.0 software (Media Cybernetics, USA) by analyzing more than six fields per tissue section.

Immunohistochemistry and immunofluorescence staining were performed to detect biomarkers of different IEC types and cell proliferation markers in intestinal sections or intestinal organoids, or β-catenin in small intestinal organoids. Briefly, intestinal sections were deparaffinized, rehydrated, and boiled in citrate solution to retrieve antigens. For immunohistochemical staining, sections were blocked with Enhanced Endogenous Peroxidase Blocking Buffer (Beyotime Biotechnology) and QuickBlock™ Blocking Buffer (Beyotime Biotechnology), followed by incubation with primary antibody overnight at 4°C and secondary antibody for 1 h at room temperature. Positive signals were developed using a DAB substrate kit (Vector Laboratories, Burlingame, CA, USA). The sections were then counterstained with hematoxylin solution. For immuno­fluorescence staining, intestinal sections were blocked with ­QuickBlock™ Blocking Buffer (Beyotime Biotechnology). For immunofluorescence staining of intestinal organoids, organoids cultured in Matrigel were isolated using a gentle cell dissociation reagent (STEMCELL Technologies, Vancouver, BC, Canada), fixed in 4% paraformaldehyde for 30 min, permeabilized with 0.3% Triton X-100 for 30 min, and blocked with QuickBlock™ Blocking Buffer (Beyotime Biotechnology). The procedure for incubation with primary and secondary antibodies in immunofluorescence staining was identical to that in immunohistochemical staining. DAPI was used to stain nuclei. The following primary and secondary antibodies were used: anti-OLFM4 (1:200; 39141, Cell Signaling Technology, Danvers, MA, USA), anti-active β-catenin (1:500; 8814, Cell Signaling Technology), anti-Ki67 (1: 500; 550609, BD Bioscience, San Jose, CA, USA), anti-BrdU (1:500; sc-32323, Santa Cruz Biotechnology, Dallas, TX, USA), anti-lysozyme (1:500; A0099, Dako, Glostrup, Denmark), anti-chromogranin A (1:200; ab283265, Abcam, Cambridge, UK), anti-GFP (1:200; M20004, Abmart, Shanghai, China), HRP-conjugated secondary antibody (1:1000, Beyotime Biotechnology), and Alexa 546 or 488-conjugated secondary antibody (1:500, Invitrogen, Carlsbad, CA, USA). Images were captured using a Revolve inverted integrated fluorescence microscope (RVL-100-G, Echo) or a high-throughput biological tissue processing and analysis system (Vectra 2, PerkinElmer, Waltham, MA, USA). Image-Pro Plus 6.0 software (Media Cybernetic) was used to quantify positive cells in intestinal sections after immunohistochemical and immunofluorescence staining. For immunohistochemical staining, six fields per section were analyzed in *Fpr2^f/f^* and *Fpr2^VKO^* mice, and more than 20 fields per section were analyzed in X-ray and 5-FU-treated mice, as well as in control mice. For immunofluorescence staining, at least 20 fields per section were analyzed. To quantify Ki67 and LYZ-positive signals in intestinal organoids, more than 30 organoids per experimental group were analyzed. More than 20 organoids per experimental group were analyzed to quantify β-catenin-positive signals in intestinal organoids.

### Intestinal organoid culture and treatment

Crypts were isolated from the small intestine as previously described [58], mixed with growth factor-reduced Matrigel (Corning, Corning, NY, USA), plated in pre-warmed 48-well plates, and ­cultured in IntestiCult™ organoid growth medium (STEMCELL Technologies, Vancouver, BC, Canada). The medium was refreshed every 2 days. To determine whether the activation of FPR2 or FPR1 is involved in organoid growth and budding, the organoids were exposed to 1 μmol/L MMK-1 (MedChemExpress, Monmouth ­Junction, NJ, USA) in the presence or absence of FPR2 antagonist WRW4 (10 μmol/L, MedChemExpress); or to 10 μmol/L fMLF (MedChemExpress) in the presence or absence of the FPR1 anta­gonist cyclosporine H (CyH, 1 μmol/L, MedChemExpress). An equal concentration of dimethyl sulfoxide (DMSO) was utilized as a vehicle control. Images of the organoids were captured on the third and fifth day of culture using a Revolve inverted integrated fluorescence microscope (RVL-100-G, Echo). The number of buds per organoid was counted using Image-Pro Plus 6.0 software (Media Cybernetic).

### Western blot analysis

Intestinal organoids were lysed, and protein concentrations were determined by BCA assay. Equal amounts of protein from each sample were then separated via SDS-PAGE, transferred to PVDF membranes, and probed with primary antibodies against FPR2 (1:1000; Novus Biologicals, Centennial, CO, USA) or HSP90 (1:1000; Abcam), followed by HRP-conjugated secondary antibodies. Target protein bands were visualized by chemiluminescence and quantified using ImageJ software (National Institutes of Health, Bethesda, MD, USA).

### scRNA-seq, data processing, and data analysis

Crypts were isolated from mouse ileum and dissociated into single cells by incubation with TrypLE Express (Thermo Fisher Scientific, Waltham, MA, USA). Cells were then stained with APC anti-EpCAM (1:200; 118213, BioLegend, San Diego, CA, USA), and IECs were sorted using an ultra-high speed flow cytometry sorter (MoFlo Astrios^EQ^, Beckman Coulter, Brea, CA, USA). The scRNA-seq library construction and sequencing were performed by OE Biotech Co., Ltd (Shanghai, China) using the MobiNova-100 microfluidic platform. Raw sequencing data were processed and aligned to the mm10 mouse reference genome using MobiVision software (version 2.1), with unique mole­cular identifier (UMI) counts summarized for each barcode. The UMI count matrix was then analyzed using the R package Seurat (version 4.0.0) [59], and low quality cells or doublets were removed based on UMI or using the R package DoubletFinder (version 2.0.3) [60]. Further processing of the scRNA-seq data, including normalization, top variable gene identification, and batch effect removal, was performed using the NormalizeData and FindVariableGenes functions in Seurat (version 4.0) and the R bachelor package (version 1.6.3) [61]. Graph-based clusters were identified according to the gene expression profile of cells using the FindClusters function in Seurat and visualized using a *t*-distributed Stochastic Neighbor Embedding (*t*-SNE) algorithm. The FindAllMarkers function was used to identify marker genes of each cluster, and cell clusters were assigned based on known markers of each IEC type. Differentially expressed genes (DEGs) were identified using the FindMarkers function. *P* value < 0.05 and |fold change| > 1.2 were used as thresholds for significant differential expression. The scRNA-seq datasets have been deposited in the GEO database under accession number GSE292802.

### Cell cycle analysis

To determine cell cycle status in scRNA-seq data, we assigned a cell cycle score to each cell using the Cyclone function of the R package scran (version 1.14.3) [62]. Cells were classified as being in G1 phase if the G1 score was > 0.5 and greater than the G2/M score, in G2/M phase if the G2/M score was > 0.5 and greater than the G1 score, and in S phase if neither score was > 0.5.

To examine the cell cycle distribution of ISCs, small intestinal crypts from *Lgr5-Fpr2^f/f^* and *Lgr5*-*Fpr2^KO^* mice were dissociated into single cells using TrypLE Express and filtered through a 40-µm strainer. The cells were fixed in 1% paraformaldehyde, permeabilized and fixed in 70% ethanol at −20°C overnight, and stained with the Cell Cycle and Apoptosis Analysis Kit (PI staining) (MedChemExpress, Monmouth Junction, NJ, USA) according to the manufacturer’s instructions. GFP^high^ ISCs were gated to detect DNA content via a CytoFlex LX flow cytometer (Beckman Coulter, Brea, CA, USA). Cell cycle distribution was analyzed using FlowJo software.

### Pseudotime analysis

Pseudotime analysis of scRNA-seq data was performed using the slingshot R package (version 1.8.0). The dimensionally reduced Seurat object was transformed into a SingleCellExperiment object using the SingleCellExperiment function. A class of cells was selected as the starting cluster for trajectory inference.

### RNA extraction and qPCR analysis

Total RNA was extracted from tissues or intestinal organoids with Trizol (Invitrogen) according to the manufacturer’s instructions and reverse transcribed into cDNA using PrimeScript™ RT reagent kit (Takara, Shiga, Japan). qPCR was performed using Power SYBR™ Green PCR Mix (Thermo Fisher Scientific) on a QuantStudio™ 6 Flex Real-Time PCR System (Thermo Fisher Scientific). The 2^−ΔΔCt^ method was used to calculate the fold change of the target mRNA using *β-actin* as an internal control. Primers used for qPCR are listed in Supplementary Table S2.

### Bulk RNA-seq and data analysis

Small intestinal organoids derived from *Fpr2^f/f^* mice were treated with 1 μmol/L MMK-1 for 5 days. DMSO at the same concentration was used as the vehicle. Total RNA was extracted using Trizol reagent (Invitrogen, CA, USA) according to the manufacturer’s protocol. RNA-seq libraries were constructed using the VAHTS Universal V10 RNA-seq Library Prep Kit (Premixed Version), following the manufacturer’s instructions. RNA-seq was performed by OE Biotech Co., Ltd (Shanghai, China) on an Illumina Novaseq 6000 platform, generating 150-bp paired-end reads. DEGs were identified based on |fold change| ≥ 2 and *P* value ≤ 0.05. KEGG pathway analysis was performed using the hypergeometric test (phyper function) in R. The resulting *P* values were corrected for multiple testing using the q value package (Bioconductor). KEGG pathways with a corrected *P* value (*q*-value) ≤ 0.05 were considered significantly enriched in the candidate gene set. Raw RNA-seq data were available at GEO (GSE310387).

### Statistical analysis

Data are expressed as mean ± SEM of at least three independent experiments. Statistical significance between two groups was determined by two-tailed Student’s *t* test or one- or two-way ANOVA with Tukey’s post-test using GraphPad Prism software (version 9.0). A *P* value less than 0.05 was considered statistically significant.

## Supplementary Material

loaf045_Supplementary_Data

## Data Availability

All data relevant to the study are provided in the article or Supplemental Information. The scRNA-seq and bulk RNA-seq datasets generated in this study are deposited in the Gene Expression Omnibus (GEO) database under accession numbers GSE292802 and GSE310387, respectively.
