# Supplementary material for: Intestinal epithelial formyl peptide receptor 2 contributes to intestinal epithelium homeostasis and injury repair by regulating intestinal stem cell and transit-amplifying cell proliferation and differentiation
Source: Life Metab. 2025 Dec 24;5(3):loaf045. doi: 10.1093/lifemeta/loaf045 (PMC13148159; doi:10.1093/lifemeta/loaf045)
Supplement: loaf045_Supplementary_Data [file loaf045_supplementary_data.zip › revised_supporting_information - tu.pdf]

Supporting Information

Intestinal epithelial formyl peptide receptor 2 contributes to intestinal epithelium homeostasis and injury repair by regulating intestinal stem cell and transit-amplifying cell proliferation and differentiation

Shuting Yu, Lele Song, Wunier, Shuyu Ouyang, Youpeng Ding, Lixing Zhan, Yi Arial Zeng, Yingying Le

This file includes:

1. Supplementary Figures S1−S11.
2. Supplementary Table S1−S2.

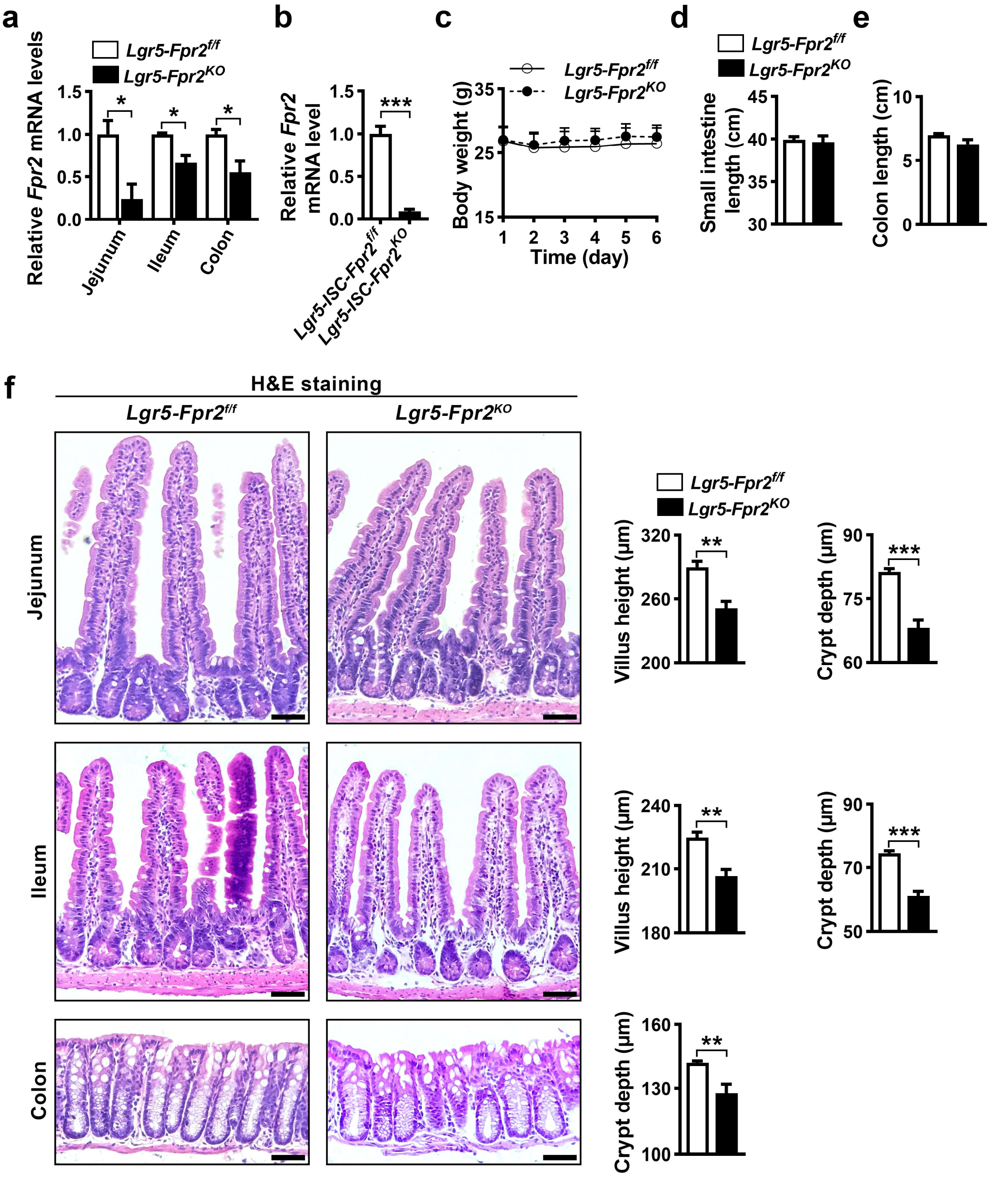

Supplementary Figure S1 *Fpr2* deficiency in mouse intestinal stem cells impairs intestinal epithelium homeostasis. (a) Expression of *Fpr2* in intestinal tissues of tamoxifen-induced intestinal stem cell-specific *Fpr2* knockout mice (*Lgr5-Fpr2*^{KO}) and control mice (*Lgr5-Fpr2*$^{f/f}$) determined by RT-qPCR analysis. $n = 3$ mice/group. (b) The expression of *Fpr2* in intestinal stem cells (ISCs) from the small intestines of *Lgr5-Fpr2*$^{f/f}$ and *Lgr5-Fpr2*^{KO} mice determined by RT-qPCR analysis. $n = 3$ mice/group. (c) Body weight of *Lgr5-Fpr2*$^{f/f}$ and *Lgr5-Fpr2*^{KO} mice after tamoxifen injection. $n = 9$ mice/group. (d and e) Length of the small intestine (d) and colon (e) in *Lgr5-Fpr2*$^{f/f}$ and *Lgr5-Fpr2*^{KO} mice after tamoxifen injection for 5 days. (f) Representative H&E staining images of jejunal, ileal, and colonic sections from *Lgr5-Fpr2*$^{f/f}$ and *Lgr5-Fpr2*^{KO} mice (left) and quantification of villus height and crypt depth (right). $n = 6$ for *Lgr5-Fpr2*$^{f/f}$ mice; $n = 5$ for *Lgr5-Fpr2*^{KO} mice in (d−f). Scale bar, 50 μm. Data are presented as mean ± SEM, $^{*}P < 0.05$; $^{**}P < 0.01$; $^{***}P < 0.001$, by unpaired two-tailed Student's *t* test.

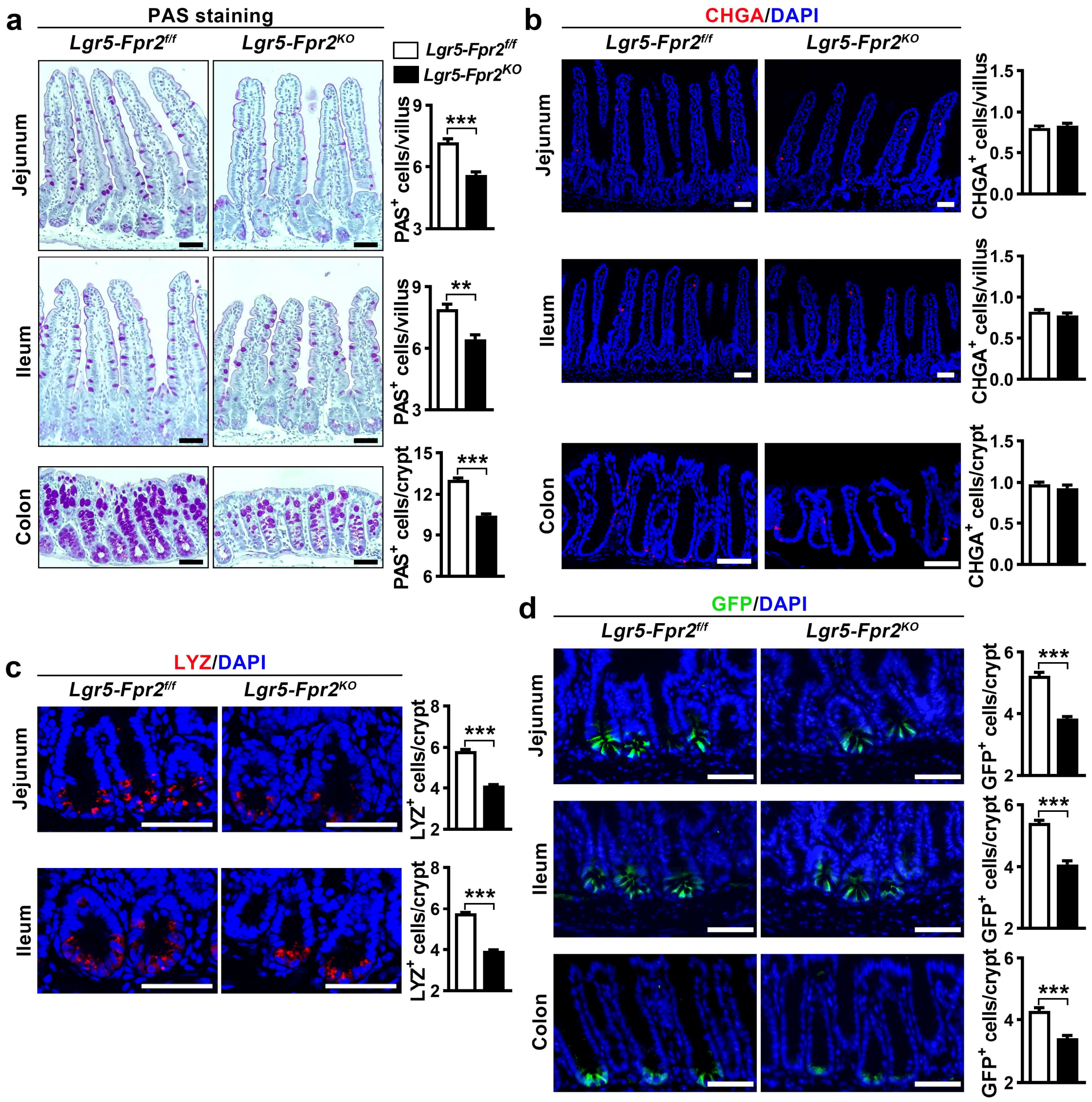

Supplementary Figure S2 Alteration of intestinal epithelial cells in intestinal stem cell-specific *Fpr2*-deficient mice. (a) Representative periodic acid-Schiff (PAS) staining images of jejunal, ileal, and colonic sections from *Lgr5-Fpr2$^{f/f}$* and *Lgr5-Fpr2^{KO}* mice, with quantification of positive cells. (b–d) Representative immunofluorescence staining images for chromogranin A (CHGA) (b), lysozyme (LYZ) (c), and GFP (d) in sections of jejunum, ileum, and colon from *Lgr5-Fpr2$^{f/f}$* and *Lgr5-Fpr2^{KO}* mice, with quantification of positive cells. n = 6 for *Lgr5-Fpr2$^{f/f}$* mice; n = 5 for *Lgr5-Fpr2^{KO}* mice. Scale bar, 50 μm. Data are expressed as mean ± SEM. $^{**}P <$ 0.01; $^{***}P < 0.001$, by unpaired two-tailed Student's t test.

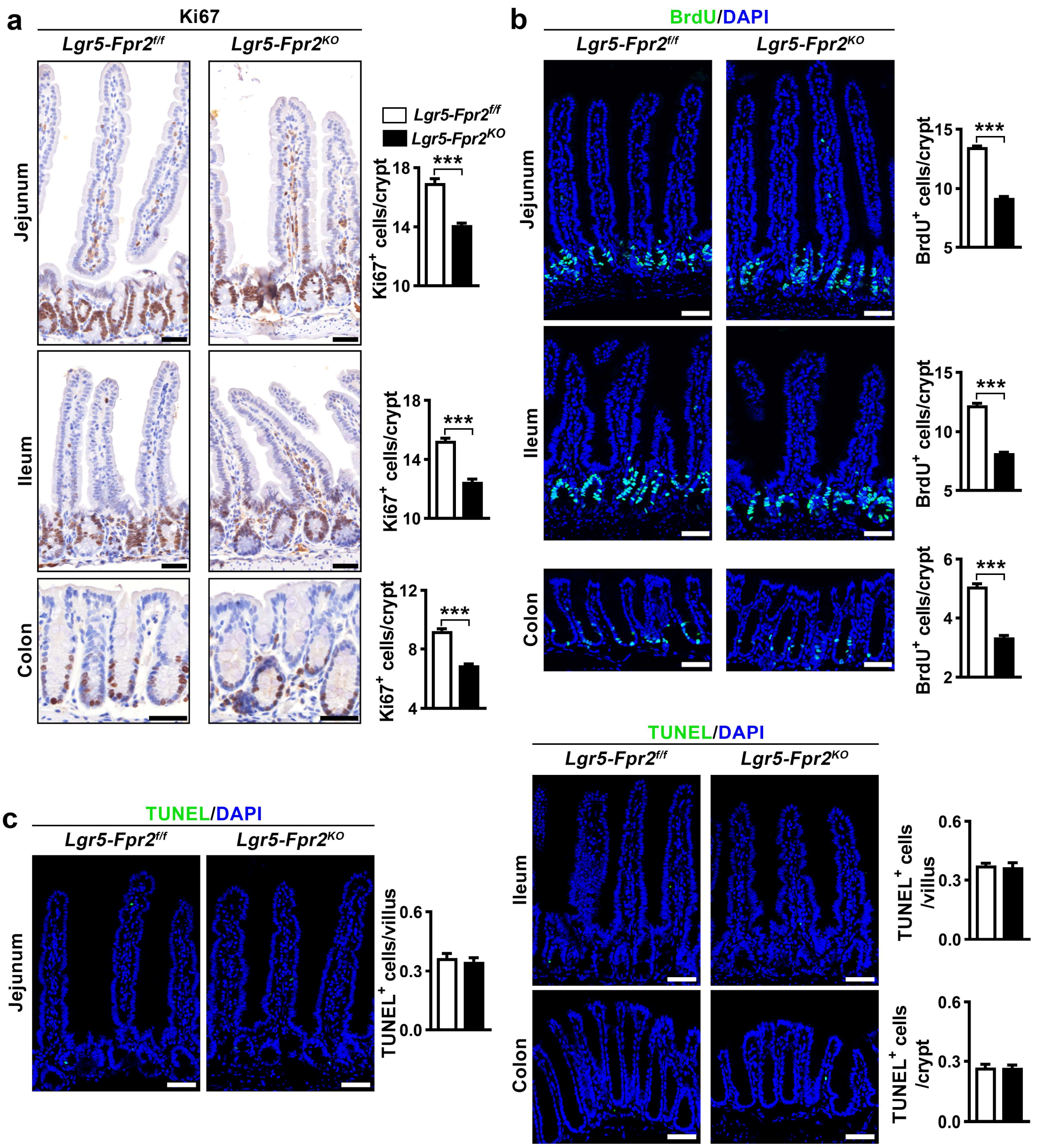

Supplementary Figure S3 *Fpr2* deficiency in mouse intestinal stem cells reduces cell proliferation in intestinal crypts. (a) Representative images of Ki67 immunohistochemical staining in sections of jejunum, ileum, and colon from *Lgr5-Fpr2*$^{f/f}$ and *Lgr5-Fpr2*^{KO} mice, with quantification of positive cells. (b) Representative immunofluorescence staining images of BrdU in sections of jejunum, ileum and colon after intraperitoneal injection of BrdU for 2 h in *Lgr5-Fpr2*$^{f/f}$ and *Lgr5-Fpr2*^{KO} mice, with quantification of positive cells. *Lgr5-Fpr2*$^{f/f}$ mice: n = 6; *Lgr5-Fpr2*^{KO} mice: n = 5 in (a) and (b). (c) Representative TUNEL assay images of jejunal, ileal, and colonic sections from *Lgr5-Fpr2*$^{f/f}$ and *Lgr5-Fpr2*^{KO} mice, with quantification of TUNEL^{+} apoptotic cells (green). n = 4 mice/group. Scale bar, 50 µm. Data are expressed as mean ± SEM, $^{***}P < 0.001$, by unpaired two-tailed Student's t test.

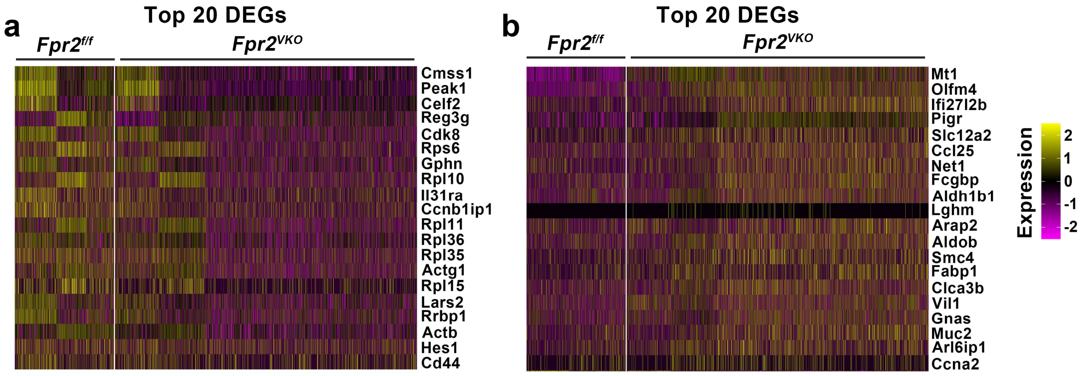

Supplementary Figure S4 Effect of intestinal epithelial *Fpr2* deletion on gene expression in immature intestinal epithelial cells. Single epithelial cells were isolated from the small intestinal crypts of *Fpr2*$^{f/f}$ and *Fpr2*^{VKO} mice for single-cell RNA sequencing. (a and b) Heatmaps showing the top 20 most significantly downregulated (a) and upregulated (b) genes, respectively, in the immature cell cluster of *Fpr2*$^{f/f}$ mice compared to *Fpr2*^{VKO} mice. n = 3 mice/group. DEGs, differentially expressed genes.

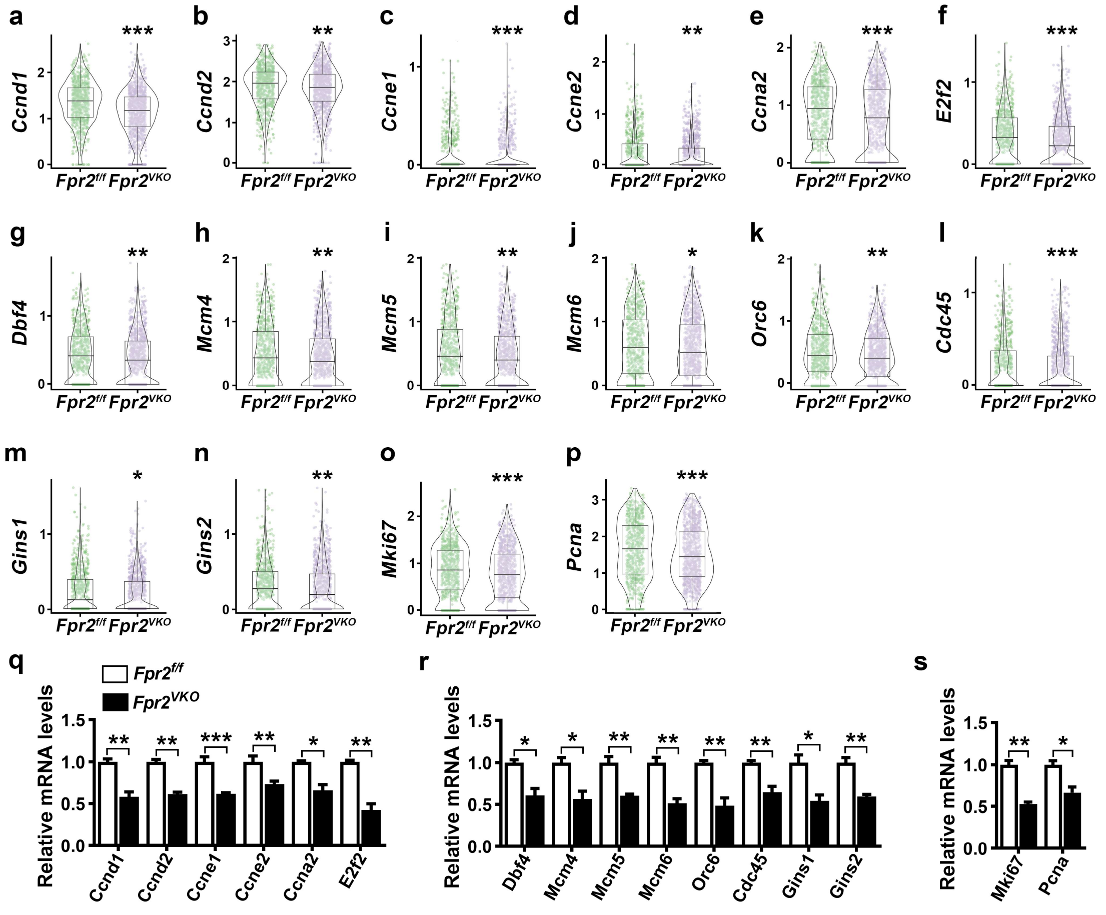

Supplementary Figure S5 *Fpr2* deficiency in mouse intestinal epithelium impairs cell cycle progression and proliferation of intestinal stem cells. Expression of genes involved in cell cycle progression and proliferation in stem cell cluster detected by single-cell RNA sequencing (a−p) and in ileal crypts detected by RT-qPCR analysis (q−s) in $Fpr2^{f/f}$ and $Fpr2^{VKO}$ mice. $n = 3$ mice/group. Data are presented as mean ± SEM, $^{*}P < 0.05$; $^{**}P < 0.01$; $^{***}P < 0.001$, by unpaired two-tailed Student's t test.

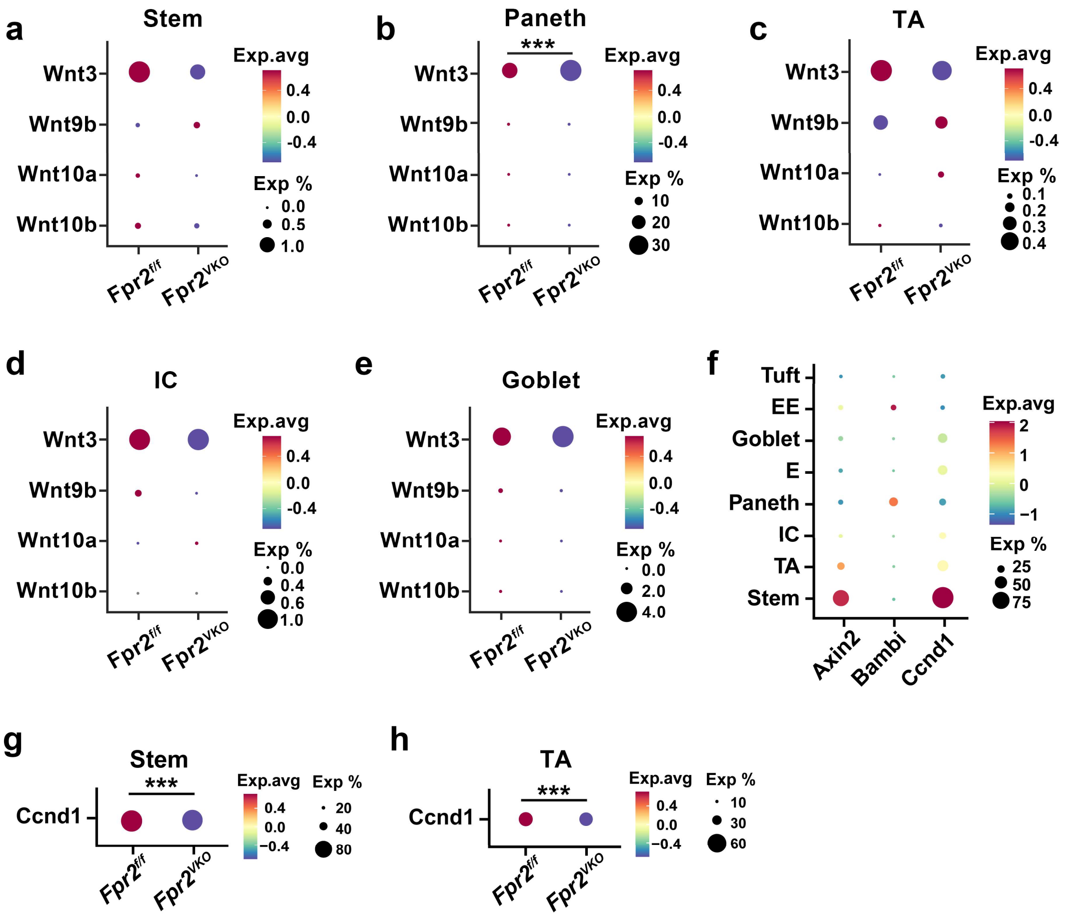

Supplementary Figure S6 Effect of intestinal epithelial *Fpr2* deletion on expression of Wnt signaling pathway genes in intestinal epithelial cells. Single epithelial cells were isolated from the small intestinal crypts of *Fpr2*$^{f/f}$ and *Fpr2*^{VKO} mice for single-cell RNA sequencing. The dot plots show the mean signature scores of the Wnt signaling pathway genes in the indicated cell clusters from *Fpr2*$^{f/f}$ and *Fpr2*^{VKO} mice. $n = 3$ mice/group. $^{***}P < 0.001$, by unpaired two-tailed Student's t test. Stem, intestinal stem cells; TA, transit-amplifying cells; IC, immature cells; Paneth, Paneth cells; E, enterocyte cells; Goblet, Goblet cells; EE, enteroendocrine cells; Tuft, tuft cells.

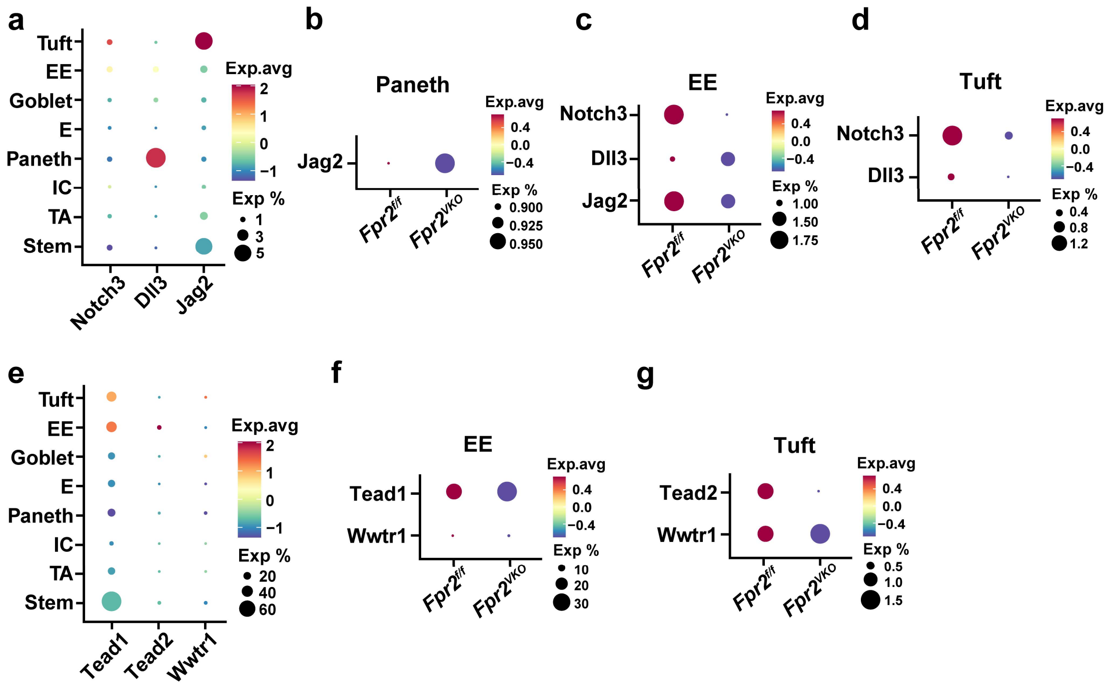

Supplementary Figure S7 Effect of intestinal epithelial *Fpr2* deletion on expression of Notch and Hippo signaling pathway genes in intestinal epithelial cells. Single epithelial cells were isolated from the small intestinal crypts of *Fpr2*$^{f/f}$ and *Fpr2*^{VKO} mice for single-cell RNA sequencing. The dot plots illustrate the mean signature scores of Notch (a–d) and Hippo (e–g) signaling pathway genes in the indicated cell clusters from *Fpr2*$^{f/f}$ and *Fpr2*^{VKO} mice. Stem, intestinal stem cells; TA, transit-amplifying cells; IC, immature cells; Paneth, Paneth cells; E, enterocyte cells; Goblet, Goblet cells; EE, enteroendocrine cells; Tuft, tuft cells.

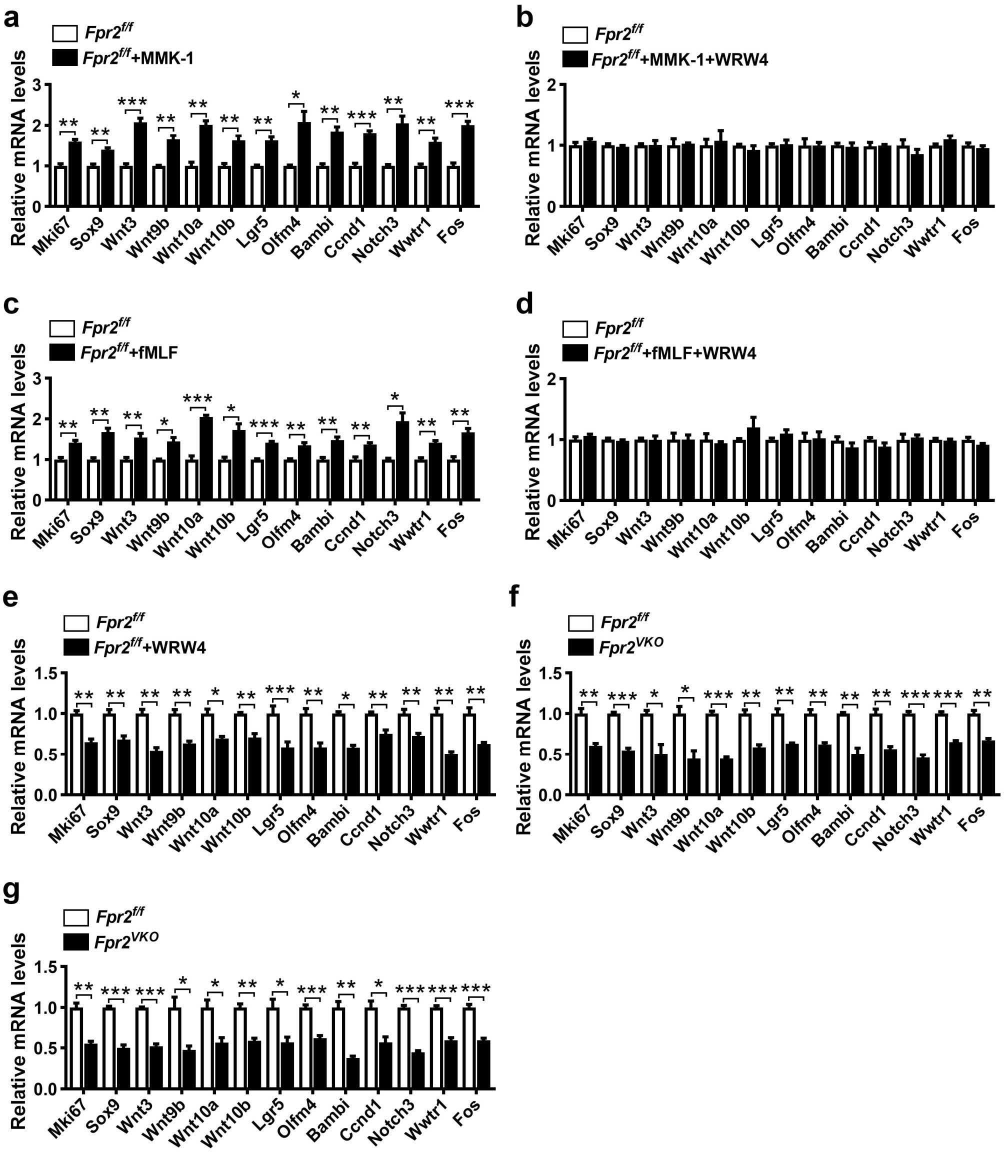

Supplementary Figure S8 Activation of FPR2 regulates the expression of genes involved in intestinal stem cell proliferation and differentiation in mouse small intestinal organoids and small intestine. (a–e) RT-qPCR analysis of the expression of the cell proliferation marker *Mki67*, the intestinal stem cell differentiation-related gene *Sox9*, and the genes involved in the Wnt (*Wnt3*, *Wnt9b*, *Wnt10a* , *Wnt10b* , *Lgr5*, *Olfm4*, *Bambi*, and *Ccnd1*), Notch (*Notch3*), Hippo (*Wwtr1*), and MAPK (*Fos*) signaling pathways, in *Fpr2$^{f/f}$* mouse small intestinal organoids treated with either 1 μmol/L MMK-1 or 10 μmol/L fMLF, with or without the FPR2 antagonist WRW4 (10 μmol/L). (f and g) RT-qPCR analysis of the aforementioned genes in small intestinal organoids (f) and small intestinal crypts (g) of *Fpr2$^{f/f}$* and *Fpr2^{VKO}* mice. $n = 3$ mice/group. Data are presented as mean ± SEM. $^{*}P < 0.05$; $^{**}P < 0.01$; $^{***}P < 0.001$, by unpaired two-tailed Student's t test.

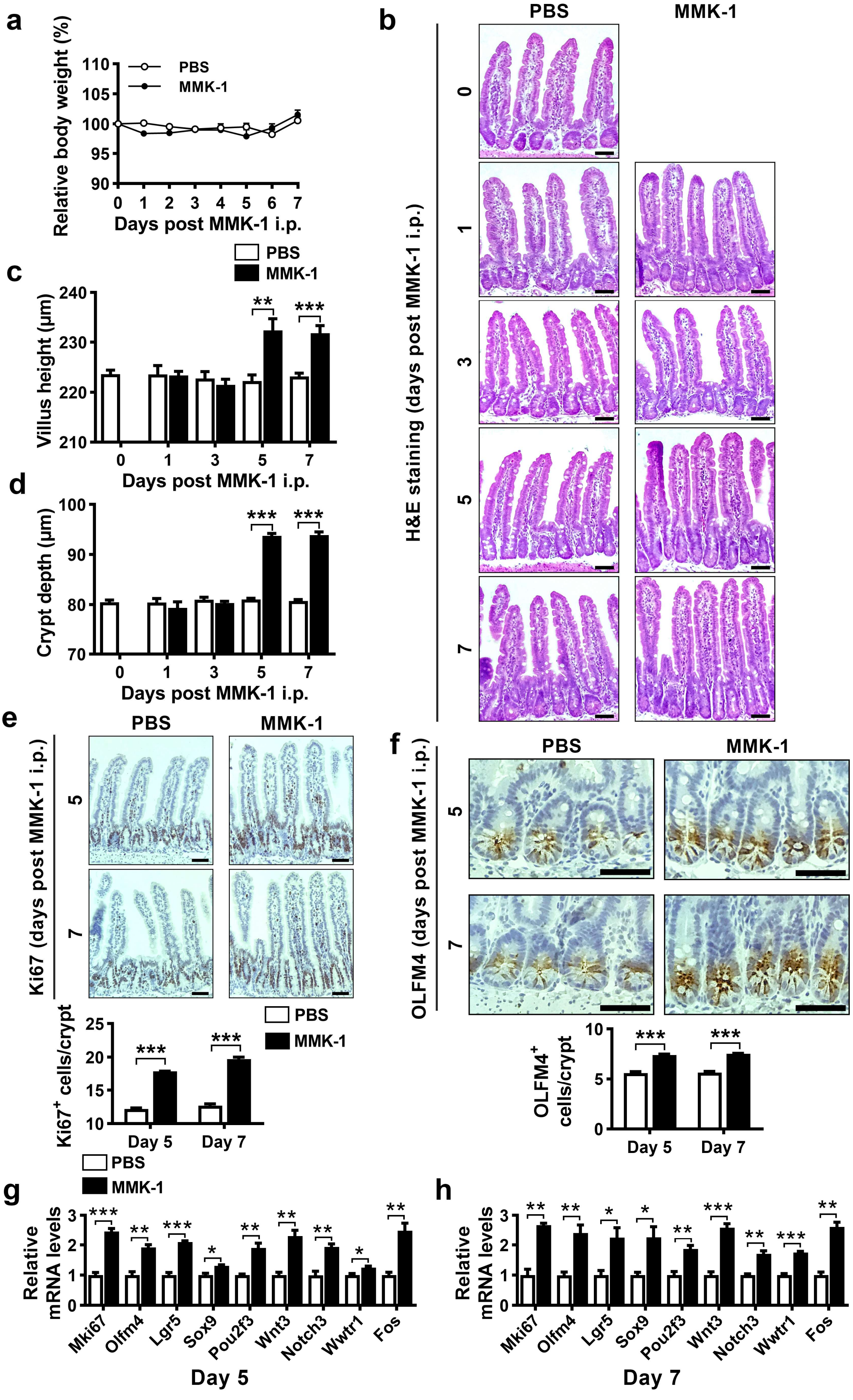

a
Relative body weight (%)
PBS
MMK-1
Days post MMK-1 i.p.
b
PBS
MMK-1
H&E staining (days post MMK-1 i.p.)
c
Villus height (μm)
Days post MMK-1 i.p.
d
Crypt depth (μm)
Days post MMK-1 i.p.
e
Ki67 (days post MMK-1 i.p.)
Ki67+ cells/crypt
Day 5
Day 7
f
OLFM4 (days post MMK-1 i.p.)
OLFM4+ cells/crypt
Day 5
Day 7
g
Relative mRNA levels
Mki67
Olfm4
Lgr5
Sox9
Pou2f3
Wnt3
Notch3
Wwtr1
Fos
Day 5
h
Relative mRNA levels
Day 7

Supplementary Figure S9 Effect of FPR2 activation on intestinal epithelium homeostasis. Mice were injected intraperitoneally with MMK-1 (10 mg/kg body weight/day) or an equal volume of PBS for 5 days. The small intestines were harvested at various time points after injection for analysis. (a) Body weight changes in mice after MMK-1 or PBS treatment. (b−d) Representative H&E staining images of small intestinal sections from PBS- and MMK-1-treated mice (b), as well as quantification of villus height (c) and crypt depth (d). (e and f) Representative immunohistochemical staining images of Ki67 (e) and OLFM4 (f) in small intestinal sections from MMK-1- or PBS-treated mice, with quantification of positive cells. n = 6 mice/group. (g and h) RT-qPCR analysis of the cell proliferation marker *Mki67*, intestinal stem cell (ISC) markers (*Olfm4* and *Lgr5*), ISC differentiation-related genes (*Sox9* and *Pou2f3*), and the genes involved in the Wnt (*Wnt3*), Notch (*Notch3*), Hippo (*Wwtr1*), and MAPK (*Fos*) signaling pathways, in the ileum of MMK-1- or PBS-treated mice. n = 3 mice/group. Scale bar, 50 µm. Data are presented as mean ± SEM. $^{*}P < 0.05$; $^{**}P < 0.01$; $^{***}P < 0.001$ by unpaired two-tailed Student's t test.

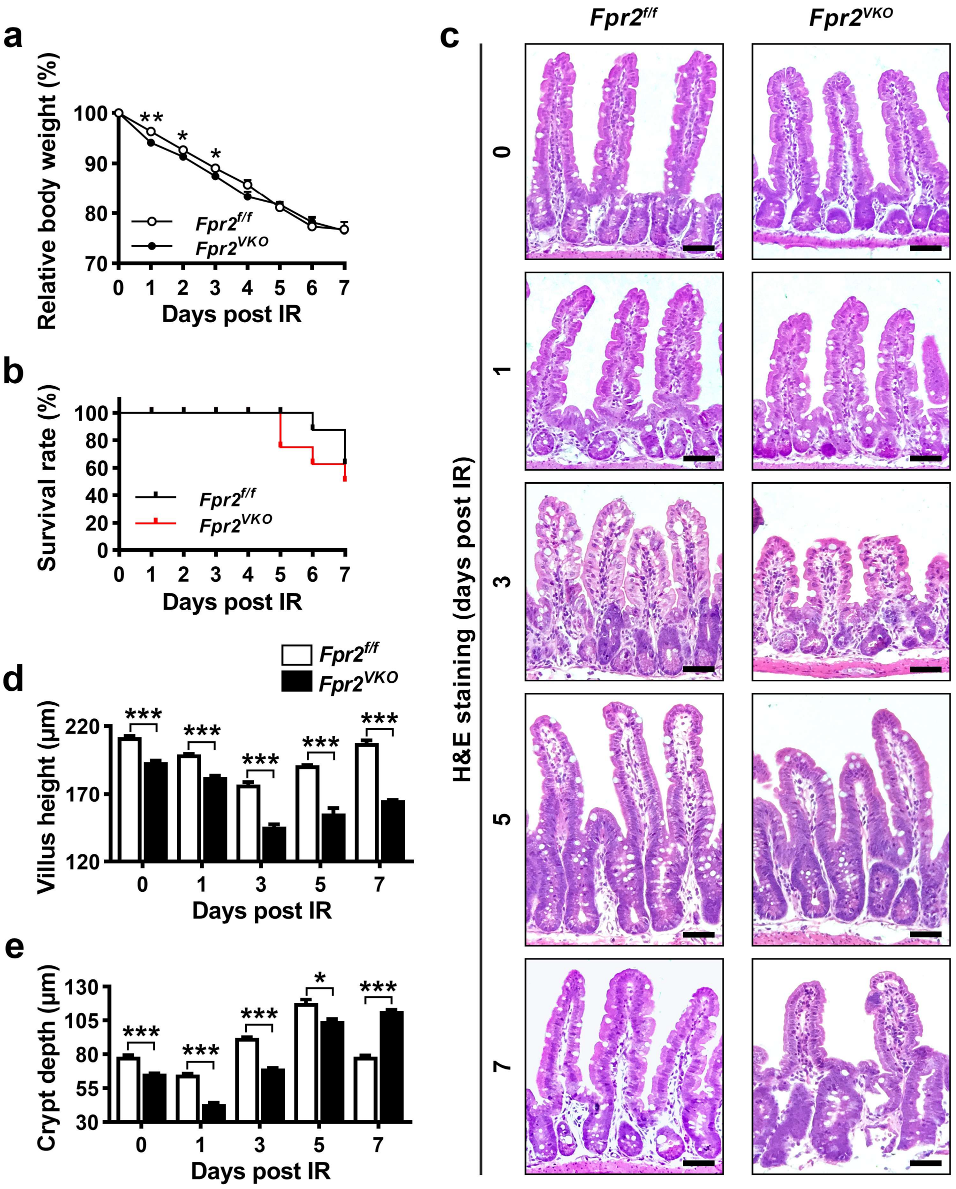

Supplementary Figure S10 *Fpr2* deficiency in intestinal epithelial cells reduces the survival rate of mice and impairs regeneration of the intestinal epithelium following injury induced by irradiation. *Fpr2*$^{f/f}$ and *Fpr2*^{VKO} mice were exposed to 10 Gy X-rays and their small intestines were harvested for analysis at various time points post-irradiation (IR). (a and b) Body weight (a) and survival rate (b) of *Fpr2*$^{f/f}$ and *Fpr2*^{VKO} mice after IR. (c−e) Representative H&E staining images of small intestine sections from *Fpr2*$^{f/f}$ and *Fpr2*^{VKO} mice after IR (c), and quantification of villus height (d) and crypt depth (e). n = 3 mice/group on days 0, 1, and 5 after IR; n = 6 mice/group on days 3 and 7 after IR. Scale bar, 50 μm. Data are presented as mean ± SEM. $^{*}P < 0.05$; $^{**}P < 0.01$; $^{***}P < 0.001$, by unpaired two-tailed Student's t test.

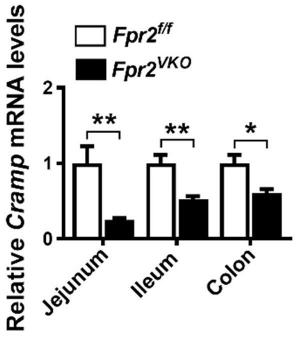

Supplementary Figure S11 Intestinal epithelial *Fpr2* deficiency reduces *Cramp* expression in the small intestine. Expression of *Cramp* in intestinal tissues of *Fpr2*$^{f/f}$ and *Fpr2*^{VKO} mice was determined by RT-qPCR. $n = 6$ mice/group. Data are presented as mean ± SEM. $^{*}P < 0.05$; $^{**}P < 0.01$, by unpaired two-tailed Student's t test.

Supplementary Table S1 Predicted FOS binding sequence in the promoter region of genes.

Gene	Predicted FOS binding sequence
Wnt3	GTGAATCA
Axin2	GTGACCCA
Sox9	GTGACTCA
Bambi	GTGATTAA
Tead1	GTGAGAAA
Tead2	GTGACCCA
Wwtr1	GTGCCTCA

Supplementary **Table S2 Primer sequences for RT-qPCR analysis.**

Genes	Primer sequences (5’→3’)
Mouse actin	F: GCTTCTAGGCGGACTGTTACT<br>R: GCCTTCACCGTTCCAGTTTTT
Alpi	F: GGTCAAGGCCAACTACAAGA<br>R: CACGGTACATCACTGAGAAGAC
Axin2	F: TGACTCTCCTTCCAGATCCCA<br>R: TGCCCACACTAGGCTGACA
Bambi	F: GATCGCCACTCCAGCTACTTC<br>R: GCAGGCACTAAGCTCAGACTT
Ccna2	F: GCCTTCACCATTCATGTGGAT<br>R: TTGCTCCGGGTAAAGAGACAG
Ccnd1	F: GCGTACCCTGACACCAATCTC<br>R: CTCCTCTTCGCACTTCTGCTC
Ccnd2	F: GAGTGGGAACTGGTAGTGTTG<br>R: CGCACAGAGCGATGAAGGT
Ccne1	F: GTGGCTCCGACCTTTCAGTC<br>R: CACAGTCTTGTCAATCTTGGCA
Ccne2	F: ATGTCAAGACGCAGCCGTTTA<br>R: GCTGATTCCTCCAGACAGTACA
Cdc45	F: GATTTCCGCAAGGAGTTCTACG<br>R: TACTGGACGTGGTCACACTGA
Chga	F: ATCCTCTCTATCCTGCGACAC<br>R: GGGCTCTGGTTCTCAAACACT
Cramp	F: GCTGTGGCGGTCACTATCAC<br>R: TGTCTAGGGACTGCTGGTTGA
Dbf4	F: AATAAGATACAGTGTCGGGTCCC<br>R: GTCCTTCTGGAAATTGGGCTC
Dll3	F: CTGGTGTCTTCGAGCTACAAAT<br>R: TGCTCCGTATAGACCGGGAC
E2f2	F: ACGGCGCAACCTACAAAGAG<br>R: GTCTGCGTGTAAAGCGAAGT
Elk1	F: TCCTGGACCTCACGGGATG<br>R: GGGTAGGACACAAACTTGTAGAC
Fos	F: CGGGTTTCAACGCCGACTA<br>R: TTGGCACTAGAGACGGACAGA
Fpr2	F: CCGTCCTTTACGAGTCCTTACA<br>R: CAGGAGGTGAAGTAGAACTGGT
Gins1	F: ATGTTCTGCGAAAAAGCTATGGA<br>R: TCACATCAGACTGGTTTTGTTCA
Gins2	F: GAGGTGGAGTTTTTGGCCGAA<br>R: GGTAAGCCGGGGTTGAAGG

Jag2	F: CTGTGCAGCGTGTTCAGTG<br>R: GTGTCCACCATACGCAGATAAC
Lgr5	F: CCTACTCGAAGACTTACCCAGT<br>R: GCATTGGGGTGAATGATAGCA
Lyz1	F: GAGACCGAAGCACCGACTATG<br>R: CGGTTTTGACATTGTGTTCGC
Mcm4	F: GAGGAAAGCAGGTCGTCACC<br>R: AGGGCTGGAAAACAAGGCATT
Mcm5	F: CAGAGGCGATTCAAGGAGTTC<br>R: CGATCCAGTATTCACCCAGGT
Mcm6	F: GCTGTTCCTAGACTTCCTGGA<br>R: CAACCAGCGTGTTTCTCTCAG
Mki67	F: ATCATTGACCGCTCCTTTAGGT<br>R: GCTCGCCTTGATGGTTCCT
Muc2	F: ATGCCCACCTCCTCAAAGAC<br>R: GTAGTTTCCGTTGGAACAGTGAA
Notch3	F: TGCCAGAGTTCAGTGGTGG<br>R: CACAGGCAAATCGGCCATC
Olfm4	F: CAGCCACTTTCCAATTTCACTG<br>R: GCTGGACATACTCCTTCACCTTA
Orc6	F: AGAAGCAGTGAACTTGGCTG<br>R: GCGGCAGTGGTGAAAAGTG
Pcna	F: TTGCACGTATATGCCGAGACC<br>R: GGTGAACAGGCTCATTCATCTCT
Pou2f3	F: CTGGAACAGTAACGTCATCCTG<br>R: AGTTCATTGCTGCTTTGGAGTT
Sox9	F: AGTACCCGCATCTGCACAAC<br>R: ACGAAGGGTCTCTTCTCGCT
Wnt3	F: AGCGTAGCAGAAGGTGTGAAG<br>R: CCAGGTGGCCCCTTATGATG
Wnt9b	F: CTGGTGCTCACCTGAAGCAG<br>R: CCGTCTCCTTAAAGCCTCTCTG
Wnt10a	F: GCTCAACGCCAACACAGTG<br>R: CGAAAACCTCGGCTGAAGATG
Wnt10b	F: GAAGGGTAGTGGTGAGCAAGA<br>R: GGTTACAGCCACCCCATTCC
Wwtr1	F: CATGGCGGAAAAAGATCCTCC<br>R: GTCGGTCACGTCATAGGACTG
